# SERS Cheminformatics: Opportunities for Data-Driven Discovery and Applications

**DOI:** 10.1021/acscentsci.5c00785

**Published:** 2025-08-05

**Authors:** Emily Xi Tan, Lam Bang Thanh Nguyen, Yubin Jin, Yan Lv, In Yee Phang, Xing Yi Ling

**Affiliations:** † Key Laboratory of Synthetic and Biological Colloids, Ministry of Education’s International Joint Research Laboratory for Nano Energy Composites, School of Chemical and Material Engineering, 66374 Jiangnan University, Wuxi, P. R. China 214122; ‡ School of Chemistry, Chemical Engineering and Biotechnology, 54761 Nanyang Technological University, 21 Nanyang Link, Singapore 637371; § Lee Kong Chian School of Medicine, Nanyang Technological University, 59 Nanyang Drive, Singapore 636921; ∥ Institute for Digital Molecular Analytics and Science (IDMxS), Nanyang Technological University, 59 Nanyang Drive, Singapore 636921

## Abstract

Surface-enhanced Raman scattering (SERS) is a powerful analytical technique offering ultrasensitive, nondestructive molecular fingerprinting. However, challenges such as spectral overlap, noise, and signal variability, especially in complex mixtures, limit its reliability and reproducibility. With increasing volumes of complex SERS data, there is a pressing need for advanced tools to manage and interpret this information. Cheminformatics amalgamates chemical knowledge with computational methods to deliver solutions for spectral preprocessing, database management, molecular modeling, pattern recognition, and multimodal data integration. This Outlook presents a vision for uniting SERS and cheminformatics to enhance the reliability of (bio)­chemical analysis and discovery. We propose a conceptual framework built upon four interconnected pillars: (1) centralized SERS databases, (2) molecular modeling for mechanistic insights, (3) machine learning (ML) for spectral analysis, and (4) automation and artificial intelligence for expanding the SERS chemical space. Together, these four pillars form a dynamic, feedback-driven system that enhances interpretability, accelerates data-driven discovery, and facilitates real-time SERS analysis. The symbiotic relationship between SERS and cheminformatics positions this integration at the forefront of data-driven chemical research with transformative applications in materials science, catalysis, biomedical diagnostics, and environmental monitoring.

## Graphical abstract

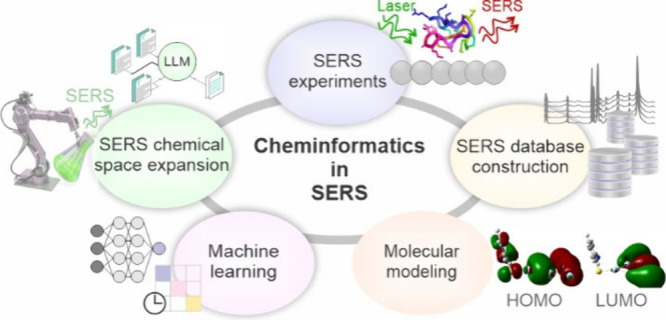

## 1 Introduction

Surface-enhanced Raman scattering (SERS) is a powerful spectroscopic technique that amplifies the weak Raman signals of molecules adsorbed on plasmonic nanostructures.^1–3^ This signal enhancement stems from localized surface plasmon resonances, which refer to the coherent oscillations of conduction electrons excited at nanostructured interfaces, typically composed of noble metals such as gold or silver.^1–3^ These resonances generate intense electromagnetic fields that boost Raman scattering intensities by factors exceeding 10^6^, producing sharp spectral peaks corresponding to molecular vibrational modes and serving as unique chemical fingerprints.^1–3^ As a result, SERS is widely employed for ultrasensitive detection of diverse molecular species, including small organics, biological analytes, environmental pollutants, and even single biomolecules such as DNA and proteins.^1–3^ In addition, the high-veracity SERS spectra not only reflect molecular structure but also respond sensitively to the local chemical environment, including surface interactions, solvation, and binding configurations.^1–7^ Overall, SERS enables ultratrace chemical detection with high sensitivity and specificity, offering qualitative and quantitative insights that make it a cornerstone of modern chemical analysis across a wide range of scientific disciplines.^8–12^

While SERS holds great promise, interpreting its data remains a significant challenge. This difficulty arises from challenges such as overlapping peaks, noise interference, and variability in signal intensity, particularly in complex mixtures or at ultralow analyte concentrations.^13–15^ Moreover, recent advances in both methodology and hardware have dramatically increased the volume and complexity of SERS data generated globally.^16–21^ This rapid growth introduces new challenges in data analysis and management, because researchers must now navigate an increasingly expanding landscape of analytes, substrates, and experimental conditions, collectively described as “SERS chemical space”, a conceptual multidimensional framework.^22^ It is clear that advanced data processing tools are critical for SERS analyses and, by extension, the broader field of analytical chemistry. These tools play a critical role in integrating data and accelerating analysis, enhancing the reliability and reproducibility of SERS results, and enabling accurate molecular detection and interpretation.

In recent years, cheminformatics has played a pivotal role in addressing the challenges of managing, analyzing, and interpreting large volumes of SERS data.^23–27^ By definition, cheminformatics integrates chemistry, computer science, and information technology and uses advanced computational methods to store, index, manage, and analyze chemical data sets. These capabilities enable researchers to extract meaningful insights from raw spectral data, tasks that were previously impossible using traditional data analysis methods.^28–30^ Currently, cheminformatics is employed for tasks such as peak identification, spectral noise reduction, multivariate statistical analysis, and pattern recognition, all of which support the identification of unknown chemicals and mixtures.^4,6,31–36^ Moreover, it facilitates the integration of SERS data with complementary techniques, such as NMR, enabling more comprehensive multimodal chemical analysis.^31,33,37–43^ The synergy between cheminformatics and SERS enhances data interpretation and accelerates real-time chemical analysis, creating new opportunities for data-driven research across diverse fields, such as fundamental chemistry, materials science, biomedical diagnostics, and food safety.^1–7^ We envision that future advances in the interface of SERS and cheminformatics will drive miniaturized, on-site and point-of-care applications, including (1) identifying unknown compounds in complex matrixes and predicting novel reactive species in reaction mechanisms, (2) facilitating rapid multiplex detection of stereoisomers and alkyl-substituted analogs, and (3) supporting targeted -omics analyses, including metabolomics, proteomics, and genomics, complementing existing gold standard liquid chromatography–mass spectrometry and sequencing tools.^4,41,44–53^

We propose a conceptual framework built upon four interconnected pillars: centralized SERS databases, molecular modeling, machine learning, and AI-driven automation to accelerate cheminformatics-driven discovery.

In this Outlook, we present a grand vision for integrating the molecular sensitivity of SERS with the advanced data analysis and pattern recognition capabilities of cheminformatics. This powerful synergy has the potential to revolutionize the way chemical data is analyzed and interpreted and also accelerate scientific progress and innovation in both fields. It unlocks new opportunities for more accurate, reproducible, and automated spectral interpretation. As a result, researchers can drive data-driven discovery, improve molecular identification, and support the development of AI systems for real-time chemical sensing and diagnostics. Our goal is to provide a conceptual framework that summarizes recent developments and outlines a forward-looking roadmap that guides future research at the intersection of SERS and cheminformatics. At the core of this integration, we propose four interdependent key pillars to advance next-generation cheminformatics-driven SERS discovery: (1) establishing a centralized SERS database, (2) leveraging molecular modeling to understand SERS data, (3) applying ML for spectral analysis, and (4) expanding the SERS chemical space via automation, robotics, and AI-driven data mining (Figure 1). Critically, we view these pillars not as isolated steps but components of a dynamic, iterative loop. Each pillar strengthens and informs the others, forming a continuous feedback loop that drives faster discovery, deeper interpretability, and greater robustness in SERS-based analytics. We begin by reviewing recent advances in how cheminformatics tools can systematically standardize, organize, and label global SERS data into a centralized database that analyzes and maps SERS spectra to chemical compounds for rapid, efficient analysis (Figure 1). Next, we explore how computational and *in silico* molecular modeling techniques transform our understanding of the SERS system by corroborating experimental findings to gain insight into the spectral data. This approach can also uncover the origins of SERS signal enhancement, paving the way for the rational design of next-generation SERS platforms (Figure 1). We then highlight the integration of SERS with cheminformatics tools, such as ML and multivariate statistical analysis, to enhance the accuracy and efficiency of complex SERS spectral analysis. This integration enables researchers to move beyond static classification and regression prediction, allowing them to tackle more advanced challenges. Specifically, it facilitates real-time dynamic analysis, driving advancement in wearable biomedical devices and environmental monitoring. Finally, we discuss emerging technologies propelling high-throughput SERS data generation, including multichannel microfluidic devices, self-driving laboratories, and large language models (LLMs) for data mining to accelerate chemical discovery (Figure 1). Together, SERS and cheminformatics form a symbiotic relationship, whereby SERS provides high-throughput and real-time molecular data, while cheminformatics effectively manages and interprets this large volume of data to enable smart data-driven chemical analysis. This multidisciplinary integration positions SERS and cheminformatics at the forefront of modern chemical analysis, where data is collected and harnessed for smarter, faster, and more impactful discoveries in environmental monitoring, biomarker discovery, and pharmaceutical research.

**Figure 1:**
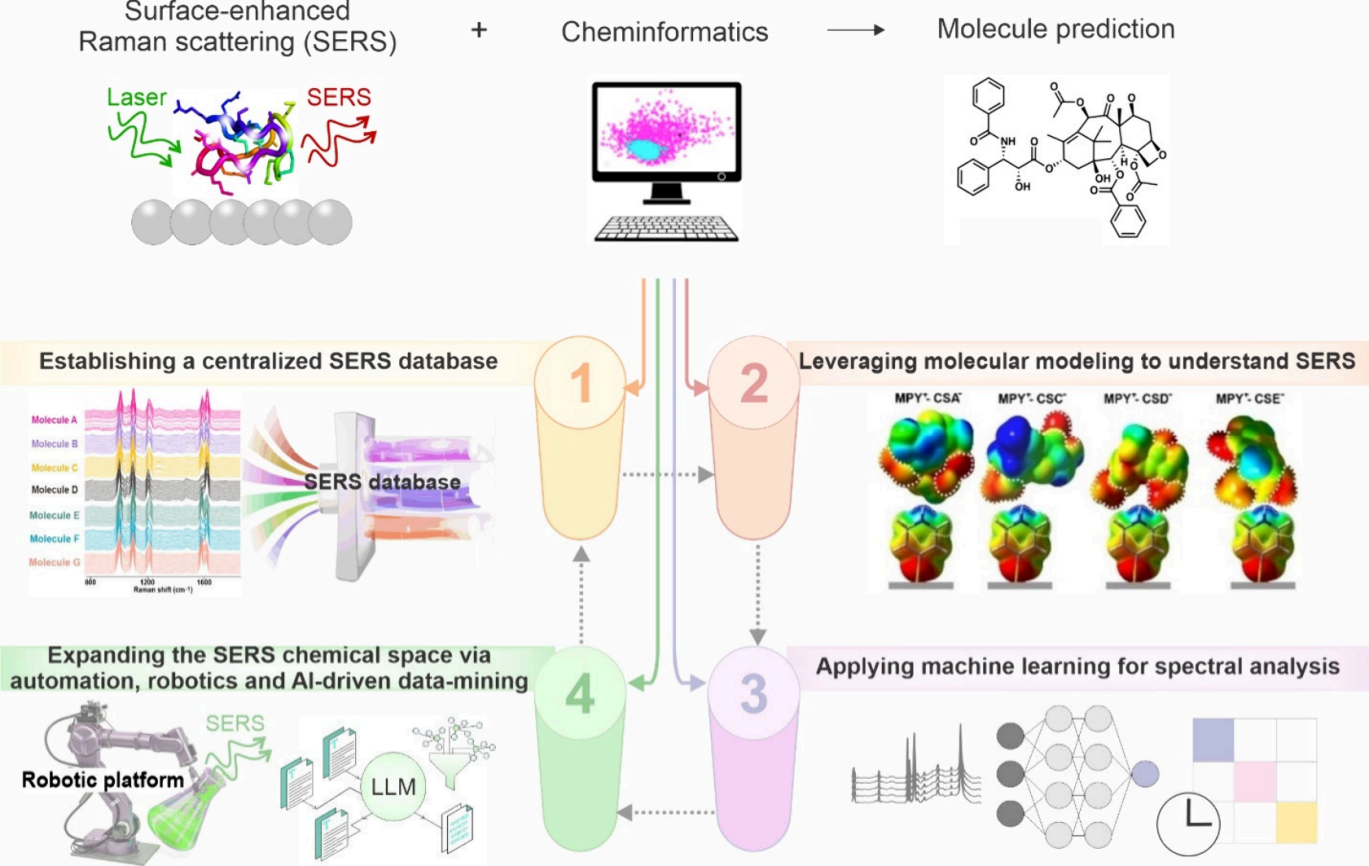
Schematics of the four key pillars reinforcing and advancing next-generation SERS-cheminformatics-based discovery. They are broadly categorized into (1) establishing a centralized and interoperable SERS database, (2) modeling molecular interactions to understand SERS phenomena, (3) applying machine learning for static and dynamic spectral analysis (Reprinted and adapted with permission from ref 7. Copyright 2023 Wiley-VCH GmbH), and (4) expanding the SERS chemical space using automation, robotics, and artificial intelligence for data mining.

Together, SERS and cheminformatics form a symbiotic relationship, whereby SERS provides high-throughput and real-time molecular data, while cheminformatics effectively manages and interprets this large volume of data to enable smart data-driven chemical analysis.

## 2 Establishing a Centralized SERS Database

Leveraging cheminformatics to establish and maintain a large-scale, centralized SERS database is essential to systematically standardize, organize, and label global SERS data.^17,23,54,55^ This approach enables the integration of broader data sets, moving beyond limited small data sets, with the ultimate goal of using this global data for more comprehensive and accurate molecular identification. We propose establishing a centralized SERS database, completed with a structured six-step framework, to account for variations in substrates, instrumentation, and measurement conditions. The six key steps are (1) data preprocessing, (2) quality control, (3) data augmentation, (4) standardized labeling, (5) data storage, and (6) data management (Figure 2A).

**Figure 2:**
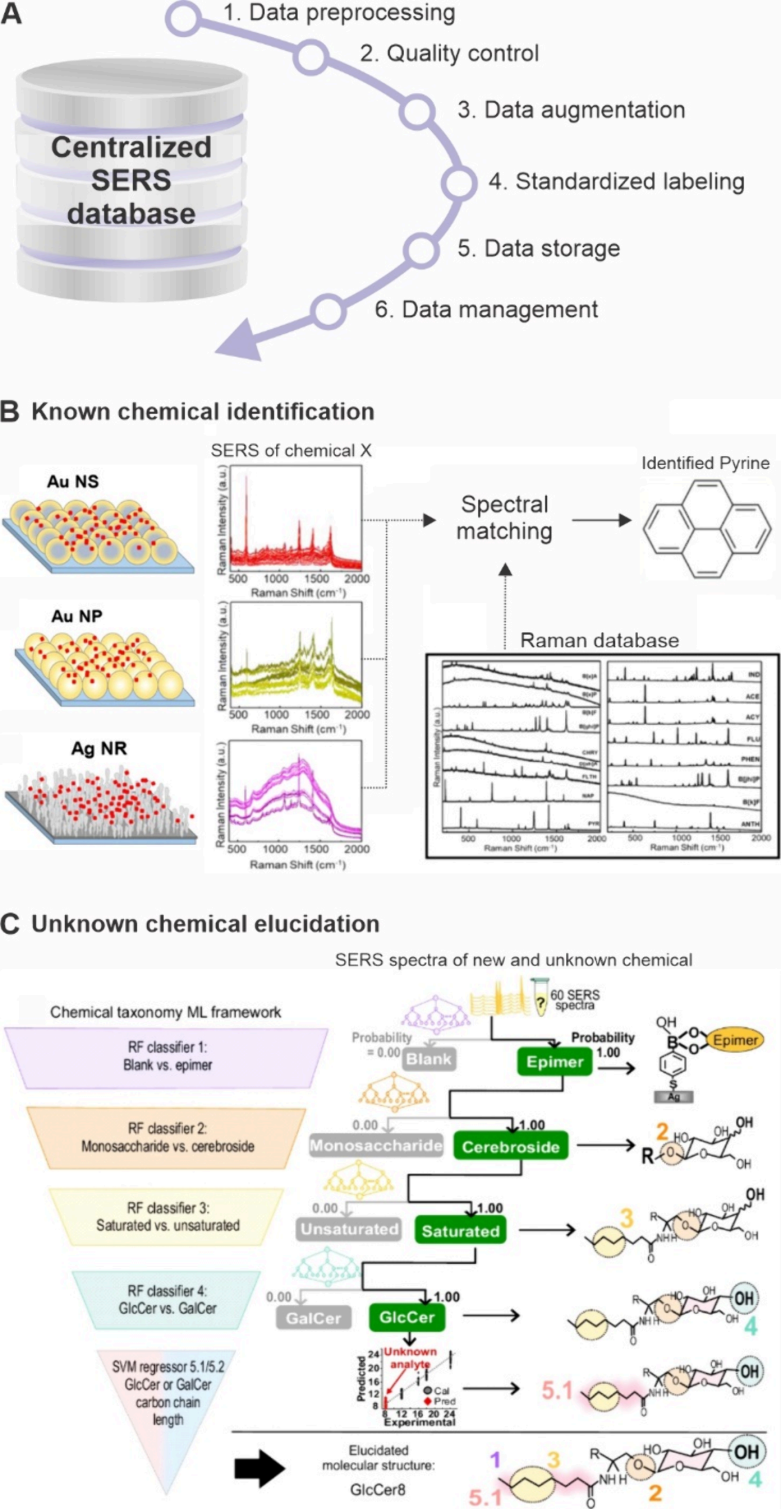
Establishing and applying a centralized SERS database to predict the molecular structures of known and unknown molecules. A. Steps to building and curating a large-scale SERS database. B. Utilizing a Raman database to identify molecules across various SERS substrates, including gold nanospheres (Au NSs), gold nanoparticles (Au NPs), and silver nanorods (Ag NRs).^37^ Reprinted and adapted with permission from ref 37. Copyright 2023 American Chemical Society. C. Predicting the structure of “unidentified” cerebroside molecules using a SERS database in conjunction with a five-level, hierarchical chemical taxonomy framework for spectral matching.^4^ Reprinted and adapted with permission under a Creative Commons CC BY 4.0 License from ref 4. Copyright 2024 Springer Nature.

A centralized SERS database offers several important advantages. First, it ensures high-quality data by incorporating the above-mentioned steps to assimilate data input from different sources and address issues such as spectral variability and sparsity. This, in turn, fosters effective data sharing and reuse across research groups. Second, it supports efficient data retrieval and robust analysis, leading to precise, reproducible, and generalizable insights that can be applied broadly across different experimental conditions. Third, such a database promotes data compatibility with various research systems, analysis tools, and platforms, promoting seamless data accessibility and integration across laboratories, institutions, and technologies. We hypothesize that such a data-driven centralized SERS database will become an important foundation for future SERS research, complementing traditional hypothesis-driven and trial-and-error approaches. Leveraging large and structured data sets can significantly enhance spectral data analysis, enabling more accurate structural elucidation and analyte quantification. This section highlights recent advancements and strategies related to these key objectives and their roles in shaping a robust and interoperable SERS data ecosystem.

The first three steps focus on improving the quality and quantity of the spectral data. Spectra data preprocessing and quality control (QC) are the first two critical steps in enhancing data quality by reducing spectral variability and ensuring consistency across experiments (Figure 2A).^56,57^ However, before any data analysis can be effective, researchers must first standardize experimentation procedures, including sample preparation, instrument calibration, and data acquisition, to ensure the reliability and comparability of SERS data.^57–59^ Spectral data preprocessing (Step 1) typically involves techniques such as baseline correction,^56^ smoothing or noise reduction,^60^ normalization,^36^ and spectral alignment.^61–63^ These methods correct systematic variations, improving signal clarity, thereby making the spectra more comparable across different experimental conditions.^57–59^ In Step 2, QC procedures involve visual inspection and signal-to-noise ratio checks for peak validation.^64^ They also include anomaly detection to identify and rectify errors, either by correcting or discarding flawed spectra,^56,60^ thereby enhancing spectral reproducibility and reliability. Step 3 focuses on data augmentation, a process aimed at increasing the quantity of spectral data. This process involves applying mathematical techniques or ML models to artificially generate synthetic variations of existing spectral data.^55,65–67^ Researchers usually apply mathematical techniques such as scaling, shifting, or spectral warping to transform data, inject noise to simulate experimental variability, and use interpolation methods to generate intermediate spectra between known samples.^66,67^ In addition, leveraging ML models such as GANs and simulations such as quantum chemical calculations, it becomes possible to predict missing spectra and simulate new experimental conditions, thereby enhancing model generalization to previously unseen data.^65–67^ These strategies mimic real-world variability and expand the training data set, which is particularly valuable when experimental data are limited or costly, thus facilitating accurate predictions in downstream applications and spectral analysis.

In addition to enhancing data quality and quantity, proper molecular labeling (Step 4), storage (Step 5), and management (Step 6) are essential to ensure efficient SERS data assimilation across laboratories, enabling quick retrieval and effective use in downstream applications (Figure 2A). In Step 4, standardized labeling refers to assigning unique and standardized identifiers to molecules associated with each spectrum. This step is critical for linking spectra to molecular structures and ensuring seamless integration with other chemical databases.^68,69^ Among these, Simplified Molecular Input Line Entry System (SMILES) stands out for encoding molecular structures as text strings based on molecular graph theory, offering computer-friendly molecular representations.^70^ Its compact, unambiguous format makes it ideal for connecting experimental data to computational models. In notable but non-SERS work, researchers used SMILES annotation to conduct a large-scale, self-learning material discovery system. They screened 7 million molecules to identify high-performance photosensitizers, four of which were subsequently synthesized, giving rise to performance matching or outperforming commercially available photosensitizers.^71^ We envision that applying SMILES to SERS will similarly facilitate efficient data identification and retrieval in a large-scale centralized SERS database. Other molecular representations, such as IUPAC International Chemical Identifier, Molecular Design Limited profiles, functionalized structure descriptors, and comprehensive general solvent descriptors, also support inputting molecular structures into ML models to predict Raman scattering properties.^32,68,69,72^ In general, standardized labeling improves the data retrieval efficiency by ensuring consistency across all entries. It also facilitates automated analysis and large-scale comparison across data sets, making it easier to uncover meaningful patterns or trends hidden in disorganized data. As a result, researchers can apply big data approaches to efficiently sift through vast amounts of data to accelerate discovery and improve spectral interpretation.^73^ We anticipate that these data-driven approaches can be applied to large-scale screening of potential SERS molecular receptors, which help to selectively bind to target analytes and enhance signal specificity.

Steps 5 and 6 focus on storing and managing data from diverse global sources while safeguarding its structure and integrity, which are crucial for building an interoperable, centralized SERS database (Figure 2A). Data storage (Step 5) refers to the physical maintenance of data, whether in databases, cloud systems, or local drives, focusing on preserving security and accessibility. In SERS, it is known that variations in experimental setups and measurement conditions can cause significant spectral discrepancies.^1,17,56,57^ To address these challenges, implementing well-defined data structures and structured storage strategies is critical for capturing standardized and detailed metadata that includes experimental conditions, substrates, instrumentation, and sample provenance.^56,74^ Organizing metadata enables researchers to better interpret and preserve the structure and integrity of large SERS spectral data sets. These practices ensure consistent, reliable data storage and enable comprehensive metadata documentation, long-term retention, and reproducibility across the centralized spectra-sharing repository.^56,74^ Meanwhile, data management (Step 6) governs the data throughout its lifecycle, preserving its usability, quality, and organization over time. It ensures data quality, accessibility, and compliance through proper version control and access management. Adopting standardized data management guidelines, including best practices in data security, ethical use, and data-sharing protocols, can protect database integrity, maintain data consistency, and ensure system reliability and long-term sustainability.^56,74^ These practices also support ethical data sharing, enable compatibility across tools and systems, helping to uphold the integrity, functionality of centralized spectral repositories^56,74^ Prior work in mass spectrometry (MS) has demonstrated the benefit of metadata inclusion and usage. For example, Reference Data-Driven (RDD) analysis was used to enhance untargeted metabolomics by matching MS/MS data with metadata-annotated source data, effectively creating a pseudo-MS/MS reference library through metadata-based source annotation.^75^ We anticipate that when establishing a SERS-based omics database, Step 4 (standardized labeling) will help identify compounds across both targeted and untargeted SERS studies, while Steps 5 and 6 will capture and manage metadata. These steps are critical for untargeted omics applications, where contextual information is essential for reproducibility and interpretation. Ultimately, we envision a cheminformatics approach to harmonize SERS results across substrates and instruments through a metadata-rich centralized database. Specifically, we outline a six-step standardized reporting protocol that captures essential metadata, such as substrate morphology, laser wavelength, and acquisition parameters, to support an interoperable SERS database that accounts for variations in instrument design and calibration. Ultimately, by adopting these best practices, the SERS community can improve data interoperability, accelerate discovery, and enhance the reliability of spectral analysis.

Ultimately, by adopting these best practices from Steps 1–6, the SERS community can benefit from improved data interoperability, accelerate discovery, and enhance the reliability of spectral analysis.

Looking ahead, for the SERS database to stay relevant and dynamic, it is important to consider implementing mechanisms for real-time updates and feedback loops. This ensures that the data are continuously enriched as new experiments, substrates, and technologies emerge in the field. Furthermore, integrating blockchain technology to track the provenance of data entries and guarantee data integrity throughout the entire process, from initial acquisition to sharing and reuse by maintaining a transparent and immutable record of the data’s history. This ensures data is reliable and verifiable, which is critical for high-stakes fields such as healthcare or environmental monitoring. Finally, ensuring the SERS database is interoperable with other chemical and spectroscopic databases (e.g., PubChem, ChemSpider) is crucial for cross-referencing SERS data with other chemical information, thereby accelerating discovery and facilitating multidisciplinary research. This cross-field interoperability will significantly enhance the value of the centralized SERS database by broadening its applicability across various research fields and enabling multimodal analysis when combined with other analytical techniques.

After establishing a centralized SERS database (Steps 1–6), researchers can leverage it for various applications, including identifying both known and unknown chemicals. For instance, one study used a standard Raman database to match and identify four polycyclic aromatic hydrocarbons from SERS spectra collected on different plasmonic substrates. By applying the Characteristic Peak Similarity (CaPSim) algorithm, the study achieved 82.4% identification accuracy (Figure 2B).^37^ This demonstrates how a standardized database can mitigate substrate-specific variability, eliminating the need for multiple substrate-specific libraries and at the same time improving scalability and generalizability in SERS-based molecular detection. While this study shows the potential of unifying the SERS database across different SERS substrates, broader unification will require careful evaluation of additional experimental variables, such as laser power, wavelength, acquisition time, and temperature. Hence, incorporating these variables into a centralized and standardized SERS database would allow researchers to share and reuse spectra across laboratories, facilitating the accumulation of a robust data set to enable accurate molecular detection.

Beyond spectral matching for identifying known chemicals, SERS databases hold great potential to advance untargeted structural elucidation, moving the field closer to the “holy grail” of identifying new and previously unknown compounds. For example, one study uses a hierarchical chemical taxonomy model to accurately elucidate the structures of unknown cerebrosides by comparing their SERS spectra to entries in a curated SERS database (Figure 2C).^4^ This approach classifies molecules through multiple levels of chemical hierarchy, such as headgroup identity, glycosidic linkages, and fatty acid chain, based on spectral similarities (Figure 2C).^4^ By leveraging the characteristic vibrational features encoded in the SERS signals, the model inferred key structural attributes of the unknown chemical, achieving over 90% accuracy in identifying core substructures and estimating alkyl chain length within a margin of fewer than one carbon chain.^4^ This example demonstrates how a well-organized SERS database not only enables spectral matching for known compounds but also guides the structural elucidation of unknown analytes through hierarchical classification strategies. Together, these studies underscore the potential of SERS databases for precise molecular identification and quantification in real-world applications, where both analyte identity and quantity are often unknown. However, current SERS databases are often sparse, with limited data on certain molecule classes or conditions. Therefore, expanding the scope of SERS studies to include underrepresented molecular systems and diverse experimental setups, such as different sample types, measurement conditions, experimental settings, spectrometer type, and laser wavelengths, is essential for building a comprehensive SERS database. One solution is to develop versatile, data-driven models trained on diverse data sets that reflect different materials and experimental conditions. This enables the model to learn a wide range of patterns and generalize across various conditions.

Collectively, these efforts contribute to the growing field of SERS cheminformatics, forming a foundation for advancing SERS to identify both known and unknown chemical species. Ultimately, a centralized SERS database can serve as a key resource for cheminformatics, enabling the SERS community to harness collective insights from a cohesive and interoperable data set for reliable and universally trusted spectral interpretations.

A centralized SERS database can serve as a key resource for cheminformatics, enabling the SERS community to harness collective insights from a cohesive and interoperable data set for reliable and universally trusted spectral interpretations.

## 3 Leveraging Molecular Modeling to Understand SERS Data

This section explores how SERS-derived molecular vibrational spectra reflect the underlying molecular structure, enabling three-way correlation between spectra features, structure characteristics (such as the types and numbers of functional groups present, carbon chain length, branching, and cyclic pattern), and molecular properties (physical, chemical, and biological).^76,77^ We begin by examining recent applications of computational approaches, such as density functional theory (DFT, for small molecular systems),^78–82^ molecular docking,^83,84^ and molecular dynamics^85^ (for large and complex macromolecular systems), in establishing the relationship between SERS spectra and molecular structure (Figure 3A). Building on this foundation, we then evaluate the potential of applying the quantitative structure–activity relationship (QSAR) approach to predict key molecular properties, including solubility, bioactivity, and toxicity. Together, these strategies enable a more comprehensive and generalized SERS prediction framework for both small and large complex molecular systems and ultimately close the loop in bridging the three-way interrelation among spectra, structure, and properties in a predictive manner.

**Figure 3:**
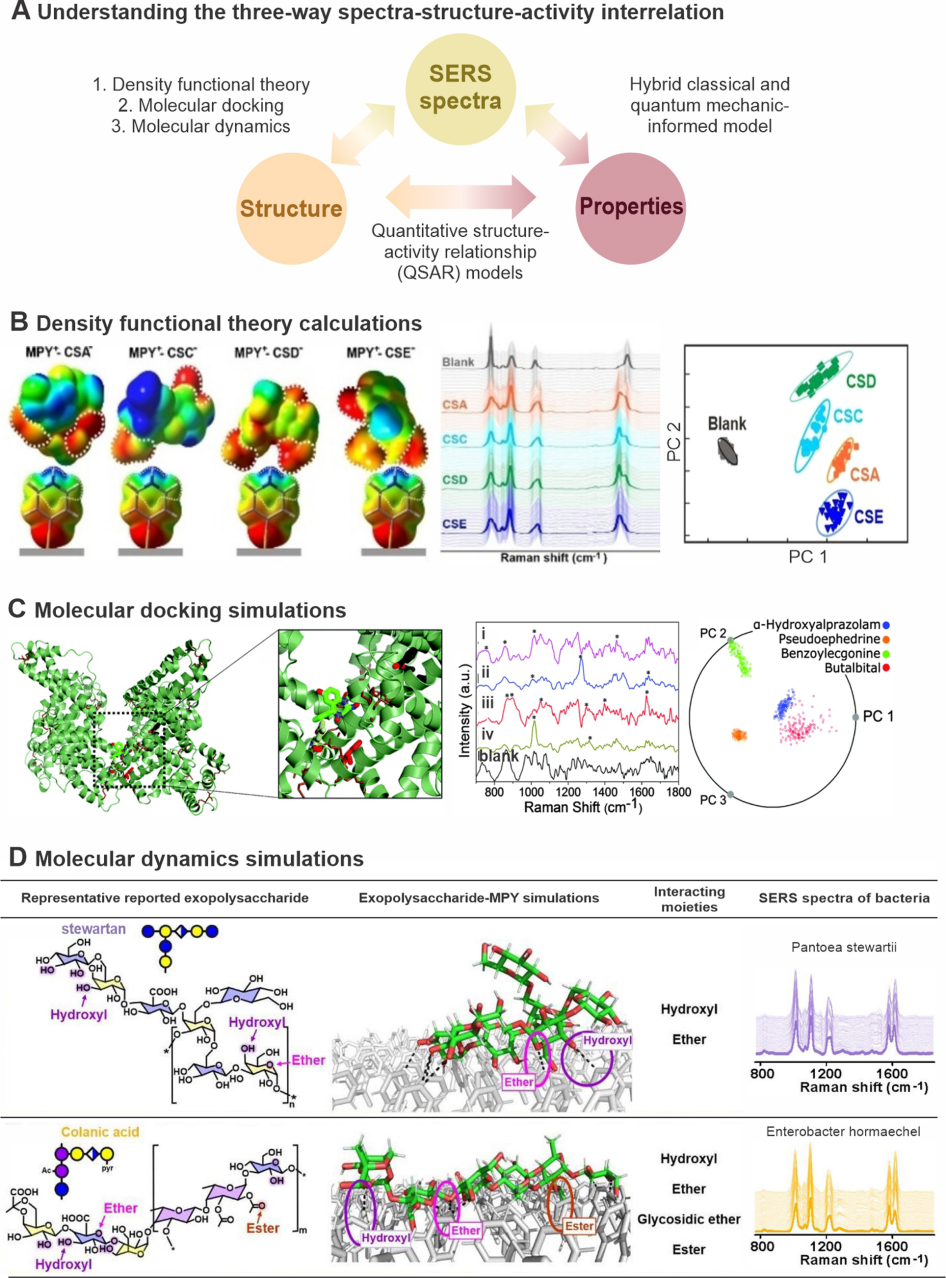
Utilizing *in silico* molecular techniques to understand SERS data. A. Schematic of establishing and understanding the three-way interrelation between SERS spectra, molecular structure, and molecular properties. B. Utilizing density functional theory to corroborate charge and geometry complementarity between the 4-mercaptopyridine (MPY) probe and four chondroitin sulfate disaccharides (CSA, CSC, CSD, and CSE) for SERS differentiation.^7^ Reprinted and adapted with permission from ref 7. Copyright 2023 Wiley-VCH GmbH. C. Molecular docking reveals drug molecules (stick representation) binding to human serum albumin induces distinct protein–drug complexes (butalbital, alpha-hydroxyalprazolam, pseudoephedrine, benzoylecgonine, labeled i–iv) and SERS spectra compared to HSA alone (blank), enabling differentiation.^52^ Reprinted and adapted with permission from ref 52. Copyright 2016 Royal Society of Chemistry. D. Using molecular dynamics (MD) simulations to examine the differences in primary interaction sites and atom contact numbers between exopolysaccharides of bacteria and a 4-MPY-functionalized Ag monolayer surface.^6^ Reprinted and adapted with permission from ref 6. Copyright 2023 American Chemical Society.

DFT uses quantum mechanical theory to calculate the distribution of electrons in molecules, materials, and surfaces, and provides molecular properties such as bond lengths, bond angles, and electronic states for studying chemical reactions and catalysis.^78^ Notably, it also enables geometry optimization and molecular vibration analysis to (1) simulate Raman spectra, (2) elucidate SERS enhancement mechanisms, and (3) guide the optimization of SERS platforms.^5,7,78,86^ First, it simulates Raman spectra, providing a theoretical benchmark against experimental data.^5,78^ In one study, the DFT-simulated SERS spectra were corroborated with experimental spectra to confirm the formation of tridentate cooperative hydrogen bonds between 6-thioguanine (TG) as a supramolecular receptor with various analytes.^5^ The DFT simulations demonstrated that the cooperative hydrogen bonds between receptor and analyte are thermodynamically stronger than the analyte–analyte interaction, which contributed to high spectra consistency. This enabled accurate identification of 16 polyfunctional analogs, including glycerol, disubstituted propane, and vicinal diols, with an accuracy of >97% using ML classification model.^5^ This synergy among experimental, computational, and ML algorithms enables researchers to identify subtle spectral trends and correlations that are challenging to discover via manual spectral analysis and to confirm the mechanism of such interaction-induced spectral trends. Next, DFT can be used to elucidate the SERS enhancement mechanism.^86^ In a separate study, combined SERS and DFT analyses showed that incorporating carbonyl groups into porous architectures enhances the SERS response of organic platforms via a chemical enhancement mechanism.^86^ As revealed by DFT modeling, this enhancement was attributed to π-extended LUMOs and variations in crystalline orientation within highly ordered nanostructured films of D­(C_7_CO)-[1]­benzothieno­[3,2-*b*]­[1]­benzothiophenes (BTBT).^86^ These mechanistic insights are key to understanding and optimizing SERS substrate properties, enabling detection sensitivity through targeted molecular engineering. Furthermore, *in silico* molecular modeling enables the development of optimized SERS substrates tailored to specific applications by elucidating the surface chemistries of plasmonic nanoparticles.^7,87^ In a notable work, DFT was used to explore the “charge and geometry complementarity” between the 4-mercaptopyridine (MPY) as SERS probe and four chondroitin sulfate (CS) disaccharides as analytes. The study showed that specific SERS fingerprints can be generated due to the site-specific interactions between the MPY probe and the CS analytes (Figure 3B).^7^ This approach achieved over 97% classification accuracy for four CS isomers and five interferences, with <3% error in multiplex quantification. This work shows how DFT can predict molecular interactions with surface structures, helping design surfaces that improve SERS specificity for complex or multiplexed samples, where detecting subtle molecular differences is key. While DFT links molecular structure to SERS spectra, its calculations are computationally costly and impractical for large and complex systems involving macromolecules with >500 atoms.^79^ Although high-performance computing (HPC) platforms, cloud-based solutions, or parallel processing algorithms can help reduce the computation costs, challenges related to scalability and efficiency still hinder the practical applications of DFT for such macromolecular systems.^88,89^

To address this limitation, molecular docking and molecular dynamics simulations, adept at handling larger systems and longer time scales, are well-suited for studying dynamic macromolecular systems over extended periods.^83–85,90^ Molecular docking searches for the best ligand fit in a receptor’s binding site by evaluating binding affinity, geometric fit, and interaction energy to predict the preferred binding pose.^83,84^ Initially used in drug discovery to identify potential drug candidates by predicting how small molecules bind to target proteins,^83,84^ molecular docking is now applied in materials research to study interactions between small molecules and larger structures, such as self-assembled monolayers on plasmonic surfaces.^91,92^ When combined with SERS technology, molecular docking facilitates the design of SERS sensing arrays to optimize receptor-analyte interaction for enhanced analytical performance.^52^ In one study, molecular docking was used to design and optimize drug binding sites and provide insights into the interactions between small drug molecule analytes and target protein receptors.^52^ By integrating these optimized receptors with SERS, the approach enhanced the identification of small drug molecules such as barbiturates, opiates, amphetamines, and benzodiazepines and achieved lower detection limits than the direct SERS method (Figure 3C).^52^ This underscores how combining molecular docking with SERS improves understanding and optimization of receptor–analyte interactions, enhancing sensitivity and specificity in drug testing and clinical diagnostics.

Molecular dynamics is another useful tool that captures the dynamic behavior of molecules on plasmonic surfaces. It offers a time-resolved perspective of interactions, including structural changes, diffusion, and other dynamic properties and processes occurring over nanoseconds to microseconds.^85,90^ Briefly, it uses classical mechanics to simulate atomic and molecular motion by solving equations of motion based on initial positions and velocities using empirical force fields commonly applied to study biomolecules (proteins, DNA), phase transitions, and material dynamics.^85,90^ While molecular dynamics are not intended for full de novo structural elucidation in unknown mixtures, it is valuable in defining structure-spectra relationships, validating candidate molecular geometries, and modeling solvation or adsorption effects that influence spectral features. In one example, molecular dynamics simulations revealed a link between bacterial identity, ECM surface characteristics, and SERS fingerprints by modeling molecular interactions at the atomic level, enabling identification of six bacterial species based on their SERS spectra with >98% accuracy (Figure 3D).^6^ This strategy offers an orthogonal taxonomic approach that does not require gene amplification or specific biomarkers, making it ideal for point-of-need applications (Figure 3D).^6^ Incorporating these ubiquitous *in silico* techniques with SERS to corroborate experimental observations adds another layer of depth to the SERS data analysis. This effectively bridges the gap between theory and experiment and offers insights into the molecular origins of spectral changes and variations. At present, DFT-calculated spectra, as well as molecular docking and molecular dynamics data, are mainly used as confirmatory tools for SERS.

However, we envisage that these calculated spectra and data can increasingly be leveraged to develop ML models for real-life SERS analysis, thereby advancing SERS technology with less reliance on extensive experimental work. For instance, a model can be trained on calculated spectra and then fine-tuned with experimental data by applying empirical frequency scaling factors and varying simulation conditions (e.g., temperature and pressure) to reduce discrepancies with experimental spectra to be used to predict the molecular structures. In a recent work, a physics-informed analytical approach that integrates DFT-simulated Raman spectra with SERS enabled accurate identification of polycyclic aromatic hydrocarbons from contaminated soil, including those lacking experimental reference spectra.^93^ Combining characteristic peak extraction (CaPE) and similarity (CaPSim) algorithms, the method overcomes challenges including spectral interference and variability, demonstrating strong agreement between simulated and experimental SERS spectra for multiple polycyclic aromatic hydrocarbons. Although the method performs well on polycyclic aromatic analytes with high Raman scattering efficiencies, it is crucial to demonstrate its generalizability to analytes with low scattering efficiencies, which are essential for real-world sensing applications. To achieve this, it is important to consider two factors. First, there is a significant domain gap in which simulated spectra are idealized and lack experimental artifacts such as noise, baseline drifts, and fluorescence, which can impair model transferability to weakly scattering analytes.^78,79^ Second, the accuracy of simulations is constrained by methodological choices (e.g., functional, basis set) and often degrades for large, flexible, or highly interactive molecules. In the context of SERS, most simulations insufficiently capture key effects such as plasmonic enhancement and molecule–surface interactions, which substantially influence spectral signatures.^78,79^ Moreover, the computational cost of high-fidelity simulations limits chemical diversity, resulting in data sets biased toward rigid molecules that do not reflect the complexity of real sensing targets.^78,79^ DFT simulations also neglect dynamic and environmental factors such as temperature and pH, which affect spectra in practice.^79^ Finally, the assumption of well-defined compositions in simulated data does not hold for complex mixtures, where spectral overlap and molecule–molecule interaction effects complicate both labeling and interpretation.^5,78,79^ These limitations highlight the need for strategies that combine simulated and experimental data using domain adaptation approaches toward generalizable *in silico*-based SERS sensing.

Once the molecular structure is determined using SERS spectra and computational tools, QSAR models can be applied to predict the chemical behaviors or properties of molecules by using numerical descriptors that represent a molecule’s physicochemical, topological, electronic, and geometric features.^94,95^ By capturing this multidimensional information, QSAR models enable reliable predictions of properties for efficient screening and evaluation of chemical compounds for research and industrial applications.^94,95^ At present, QSAR models employ multivariate statistical techniques, e.g., multiple linear regression and partial least-squares (PLS), or ML algorithms, e.g., random forest and neural networks, to correlate molecular descriptors with chemical behaviors or properties.^94,95^ By integrating QSAR models with SERS and computational tools to establish the spectra-structure-properties relationship, researchers can explain experimental observations and predict critical molecular properties, such as solubility, toxicity, reactivity, and bioactivity, crucial for downstream SERS applications^94,95^ This integration enhances SERS’s interpretability and transforms SERS into a predictive and versatile tool, while enriching cheminformatics with robust new data and opportunities to probe molecular interactions.

Looking ahead, we envision that predictive models can directly infer molecular or material properties from spectral data (e.g., Raman or SERS spectra) without relying on intermediate steps (e.g., molecular simulations or QSAR modeling). This leap requires the development of more sophisticated modeling frameworks that go beyond empirical correlations and incorporate deeper physical insights. Specifically, a hybrid classical–quantum mechanical model that integrates classical and quantum mechanical principles will be essential to capture the complexity of the underlying molecular systems.^96^ The proposed hybrid model must capture the full spectrum of molecular interactions relevant to SERS, including electromagnetic interactions and chemical interactions, such as charge transfer between the analyte and substrate and environmental influences, such as solvent effects or temperature. Accurately modeling these factors in hybrid models helps avoid oversimplified views of SERS mechanisms, which are inherently complex and context-dependent, enabling reliable diagnostics, material screening, and property prediction.

## 4 Applying Machine Learning (ML) for Spectral Analysis

This section highlights how ML significantly enhances SERS analysis by providing advanced mathematical and computational tools to build predictive models and automate the processing of large-scale SERS data. This ML-driven approach accelerates and automates spectral analysis, enabling real-time prediction and minimizing human errors, offering clear advantages over traditional manual SERS analysis. Notably, SERS data can be in the form of (1) static, where it does not change once recorded or stored, or (2) dynamic, where it changes over time. We spotlight how ML algorithms, including classification, regression, and clustering methods, can identify patterns and correlations across multiple spectral variables to enhance prediction accuracy and efficiency.^97–99^ This is particularly valuable in point-of-care biomedical applications, where both classification and quantification of analytes are critical.

For example, a partial least-squares (PLS) classification model was used to analyze SERS spectra from 501 breath samples to differentiate COVID-19 positive and negative individuals. The model achieved >95% sensitivity and specificity by identifying subtle spectral variations caused by the differences in key breath-based metabolites, enabling rapid, noninvasive detection of COVID-19 under 5 min. This demonstrates the potential of ML-enhanced SERS for real-time disease detection in compact and hand-held diagnostic platforms (Figure 4A).^34^ In another work, a PLS regression model was applied to SERS spectra to simultaneously quantify two miscarriage-related urine metabolites, 5β-pregnane-3α,20α-diol-3α-glucuronide, and tetrahydrocortisone. The model effectively captured the metabolite-specific spectral features, and when benchmarked with a calibration curve, it allowed accurate quantification of the multiplex metabolites in urine samples. This approach was validated in a case-control study involving 40 patients, demonstrating its potential for predicting miscarriage risks through multiplexed metabolite profiling and quantification. (Figure 4B).^35^ Another application is fundamental catalysis research, where understanding reaction mechanisms is crucial. For instance, a regression model can link Raman spectral features to key interaction properties, such as adsorption energy and charge transfer, between metals (e.g., pristine Au or Ag substrates) and adsorbates (e.g., CO or NO). These relationships can be further generalized to predict behaviors on a wider range of metal or alloy surfaces.^100^ This data-driven approach enhances the understanding of reaction mechanisms, reaction dynamics, and catalyst performance, enabling efficient catalyst screening and reaction screening. These examples showcase the utility of ML models in the qualitative and quantitative analysis of static SERS data sets that enable rapid, noninvasive diagnostics and SERS as a point-of-care analytical tool.

**Figure 4:**
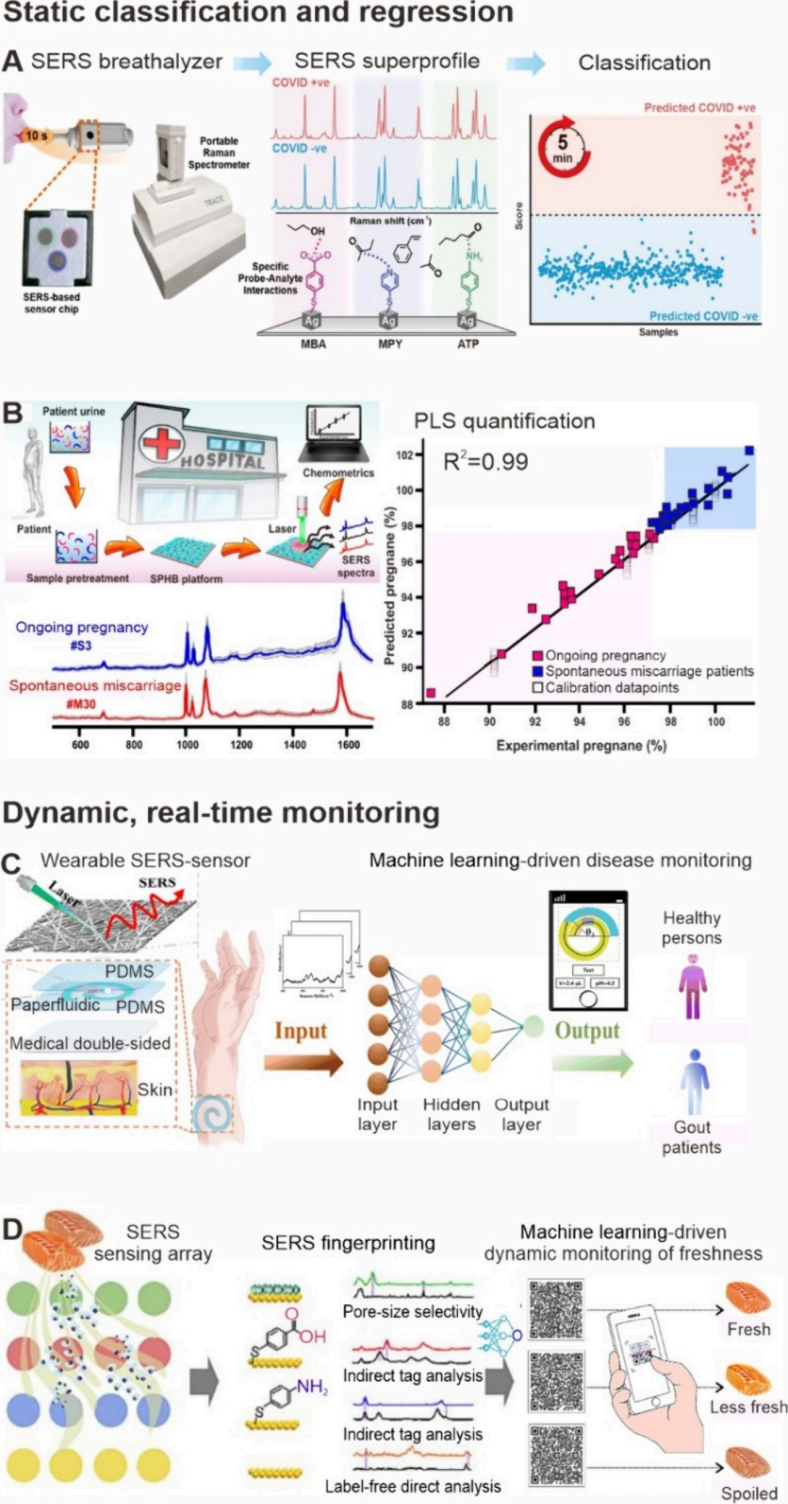
Harnessing machine learning and cheminformatics tools for static and dynamic SERS spectral data analysis. A. A classification model processed SERS spectra from 501 breath samples, achieving over 95% sensitivity and specificity for fast, noninvasive COVID-19 detection in less than 5 min.^34^ Reprinted and adapted with permission from ref 34. Copyright 2022 American Chemical Society. B. A regression model rapidly quantified key urine metabolites for miscarriage screening and pregnancy outcome prediction in a 40-patient case-control study.^35^ Reprinted and adapted with permission from ref 35. Copyright 2020 American Chemical Society. C. A wearable sweat analysis platform combining SERS, an artificial neural network, and smartphone integration enables real-time, noninvasive gout diagnosis by accurately detecting uric acid and measuring sweat pH and volume.^101^ Reprinted and adapted with permission from ref 101. Copyright 2024 Royal Society of Chemistry. D. Multidimensional chemometrics for *in situ*, real-time SERS gas sensing of multiple food-borne VOCs, including hydrogen sulfide, aldehyde, and amines.^102^ Reprinted and adapted with permission from ref 102. Copyright 2024 Elsevier.

As SERS technology advances, there is a growing need to integrate it with advanced ML to continuously process dynamic and time-dependent SERS data and enable adaptive response mechanisms. This is because real-time SERS monitoring generates large volumes and complex spectral data, making manual analysis challenging, and interpretation difficult and time-consuming. These hurdles slow the extraction of insights and impede timely decision-making in time-sensitive applications, where rapid responses are critical. Thus, automating this process with cheminformatics is crucial for delivering real-time feedback. This capability is particularly valuable in wearable SERS-based biomedical devices, which require rapid detection and continuous monitoring of metabolites in biomatrixes, such as tears and sweat, for effective health monitoring. In one study, multivariate statistical analysis was integrated with a wearable plasmonic paper-based microfluidic system, enabling real-time, continuous, and label-free detection of uric acid via SERS. The system continuously monitors sweat loss, sweat rate, and the concentration of analytes in sweat and quantifies uric acid at both physiological and pathological levels of 1 μM, by analyzing spectral features corresponding to the molecule’s vibrational modes, offering actionable insights from biochemical information for disease diagnosis and management.^103^ Another study combined a wearable SERS platform for sweat capture with ML for image recognition, analyzing both SERS and colorimetric signals. This system achieved 97% accuracy in real-time smartphone-based health monitoring, enabling noninvasive gout diagnosis (Figure 4C).^101^ By synergizing SERS spectral data analysis with visual signal processing, the SERS-colorimetric platform provided accurate diagnostic results, demonstrating its potential for rapid point-of-care disease monitoring. Such dynamic and real-time processing of complex real-life SERS data, recording biomolecular changes or physiological conditions in biofluids, is crucial for wearable devices to ensure that actionable insights or alerts are delivered without delay. Moreover, this approach can also be extended to the remote, online SERS-based identification and quantification of gaseous pollutants or food contaminants. The integration of real-time spectral monitoring facilitates the continuous detection of trace-level contaminants, enabling real-time intervention and informed decision-making, which are critical for environmental and food safety management. For example, ML was applied to enable real-time, in situ evaluation of food freshness, leveraging SERS’s ability to detect and quantify multiple food-borne volatile organic compounds (VOCs), such as bacterial metabolites, hydrogen sulfide, aldehydes, and biogenic amines (Figure 4D).^102^ This system demonstrated high sensitivity and the ability to detect even submicromolar concentrations of these VOC, providing an effective tool for food safety and quality control. We envision that fast-responding ML-driven SERS gas sensors could be applied to hazardous materials scenarios where rapid toxic gas diffusion and spread occur to provide real-time feedback and critical early warnings that prompt preventive actions. This is also beneficial in remote areas inaccessible to humans, whereby a compact Raman spectrometer can be integrated with a drone-like system for real-time air surveillance. Overall, ML enables efficient static and dynamic SERS data analysis, such as identifying subtle spectral features and detecting low-concentration analytes, with greater reliability for diverse applications. Moreover, the effectiveness of these models can be assessed from two perspectives: (1) a data-driven approach, which evaluates feature importance through ML interpretability techniques, and (2) a domain knowledge-driven approach, which cross-validates ML predictions with molecular simulations to enhance reliability.

Currently, real-time SERS data processing and analysis still face limitations, including high latency (due to the need for data to travel over long distances) and delays in handling continuous data streams, both of which hinder system responsiveness. One effective solution is edge computing, which processes data near its source to reduce latency and minimize bandwidth requirements. By transmission of only processed or summarized data to central systems, this approach can improve overall efficiency. In addition, high-performance data streaming frameworks, such as Apache, Kafka or Flink, enable fast and continuous processing of real-time data, allowing applications to react quickly to new information.^104^ Improvements to network infrastructure, such as improving data flow between systems and implementing data compression or load balancing, can reduce data transmission delays and support real-time processing.^104^ Scalability is another significant issue as systems often struggle to handle increasing data volumes and speed. In this aspect, cloud-based solutions with autoscaling capabilities and distributed computing frameworks such as Hadoop or Spark can potentially address this limitation.^88,89,105^

## 5 Accelerating the Expansion of the SERS Chemical Space

Traditionally, SERS spectra are collected in a batch manner, leading to discontinuous data acquisition and time loss during sample exchanges. This method significantly hampers the data collection efficiency. This manual data collection further limits reproducibility and scalability, which are key drawbacks in leveraging large volumes of high-quality data that are critical for cheminformatics applications. To overcome these challenges and advance the field, there is an increasing necessity to expand SERS databases and, by extension, AI-assisted SERS chemical space. We envision that this expansion will be driven by two strategies (1) generating new data using high-throughput experimentation platforms and automated data collection processes, as well as (2) using artificial intelligence (AI) to mine and extract data from existing literature, creating a more comprehensive and dynamic SERS chemical space (Figure 5A). Our vision redefines SERS from a measurement technique into a data-centric platform, where autonomous experimentation, cheminformatics, and LLM-driven mining converge to expand the chemical space at unprecedented speed and precision. In this section, we frame automation and AI-driven data mining as a unified pillar, reflecting their synergistic role in expanding the chemical space accessible by SERS. Automation generates high-throughput, reproducible spectral data, while AI mines existing data sets, identifies patterns and gaps, and guides future experiments. We emphasize that their value is maximized in a tightly integrated, closed-loop discovery framework, where each reinforces the other to enable scalable, data-centric chemical discovery.

**Figure 5:**
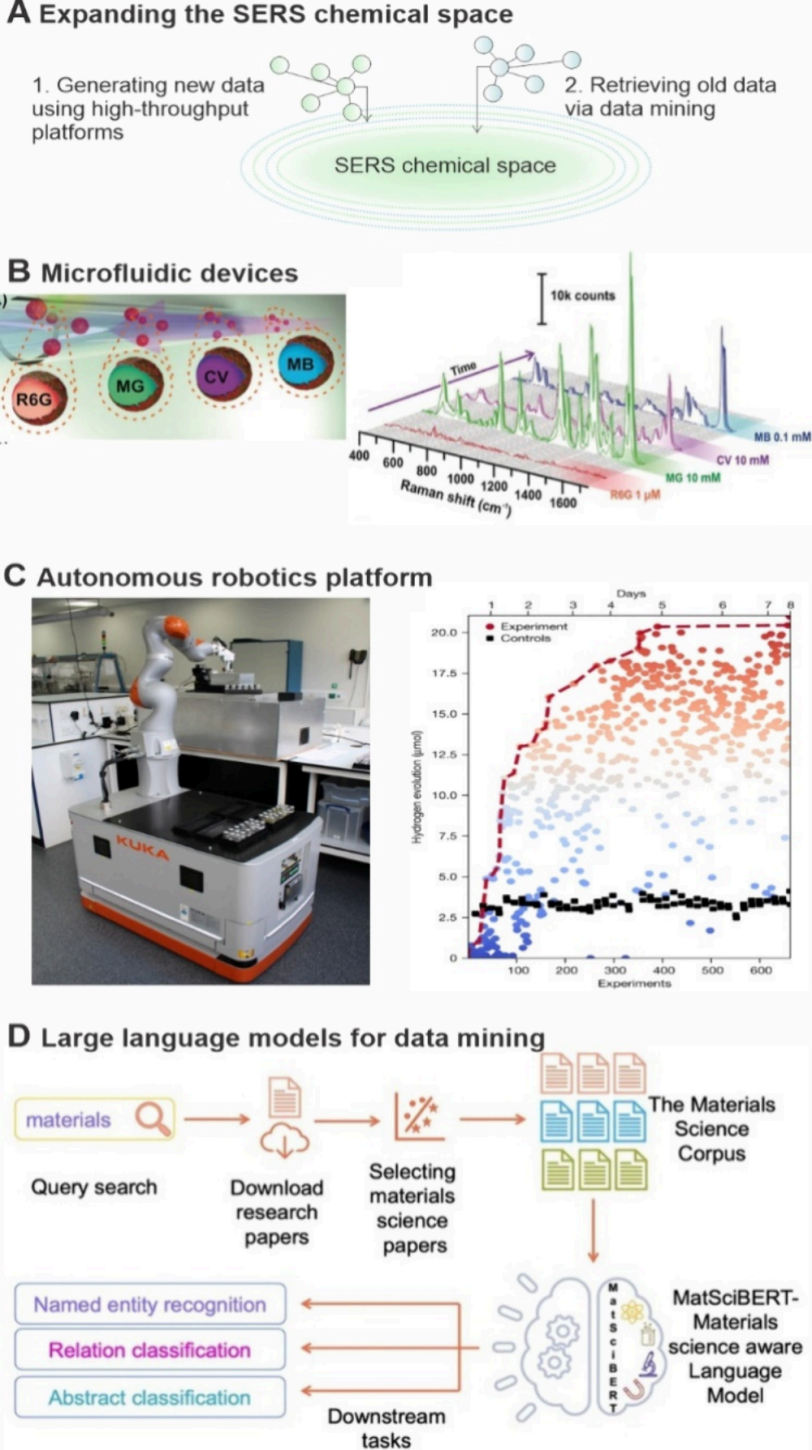
Expanding the SERS chemical space using high-throughput platforms and data mining. A. Schematic of using high-throughput experimental platforms to generate new SERS data and utilize data mining tools to retrieve old SERS data. B. Microfluidic platform for continuous flowing colloidosomes for sequential high-throughput and online SERS analysis and data collection of multiple analytes.^16^ Reprinted and adapted with permission from ref 16. Copyright 2017 Wiley-VCH GmbH. C. A mobile robotic chemist autonomously searches for hydrogen evolution catalysts, with each batch consisting of 16 experiments and 2 baseline controls. After 688 experiments, the maximum hydrogen evolution rate reached 21.05 μmol h^–1^, while the baseline was 3.36 ± 0.30 μmol.^106^ Reprinted and adapted with permission from ref 106. Copyright 2020 Springer Nature. D. The LLM MatSciBERT was used to identify and extract relevant research papers, building a comprehensive materials science corpus (MSC) library.^107^ Reprinted and adapted with permission under a Creative Commons CC BY 4.0 License from ref 107. Copyright 2022 Springer Nature. This library was evaluated across various downstream tasks, such as entity recognition, relation classification, and abstract classification, to accelerate materials discoveries.

Our vision redefines SERS from a measurement technique into a data-centric platform where autonomous experimentation, cheminformatics, and LLM-driven mining converge to expand the chemical space at unprecedented speed and precision.

In this aspect, multichannel microfluidic devices address this challenge by enabling parallel, continuous SERS experiments and the analysis of multiple samples under varying conditions. This methodology drastically reduces the time required to generate large data sets and minimizes human error. In one study, integrating 3-dimensional plasmonic colloidosomes into a microfluidic channel for online SERS detection enables continuous, multiplex detection of 20 samples in under 5 min (Figure 5B).^16^ The microfluidic channel generates isolated colloidosomes to prevent cross-sample and channel contaminations (Figure 5B).^16^ This configuration enabled accurate quantification of individual colloidosomes over a concentration range of 5 orders of magnitude, while preserving the Raman features in mixtures, allowing multiplex identification and quantification of various analytes. We envision that microfluidics can be increasingly incorporated to enable high-throughput SERS data acquisition, especially when large volumes of data are needed. Recent advancements in automated microfluidic systems, often integrated with robotics, allow precise handling of tiny fluid volumes (microliter to nanoliter range). This allows for rapid and efficient delivery of samples to SERS-active surfaces, reducing sample processing times and minimizing human interferences.^108^ These platforms provide precise control of conditions including temperature and reagent concentration, allowing systematic exploration without modifying the instrument setup. This flexibility allows diverse analyte testing and scalable data collection under various measurement conditions, helping to produce high-quality spectral data sets needed to train ML models and push the boundaries of molecular detection. Overall, these examples illustrate how integrating microfluidic systems with SERS enables rapid, simultaneous, and high-resolution spectral acquisition from multiple samples. This is invaluable for multiplexed sensing and high-throughput screening of large compound libraries while minimizing variability and enhancing reproducibility to improve data quality and quantity toward rapid SERS data generation for cheminformatics.

Alternatively, self-driving laboratories that combine robotics and AI can automate data generation by performing experiments, analyzing data, and optimizing experimental conditions without manual intervention, which helps to streamline and accelerate the SERS workflow. A notable non-SERS example is a mobile robot that autonomously synthesized photocatalysts for hydrogen production, conducting 688 experiments across ten variables to identify photocatalysts six times more active than the originals (Figure 5C).^106^ Inspired by such advances, we envision similar autonomous platforms being applied to SERS to boost the throughput of SERS substrate fabrication and measurement. By integrating rapid characterization techniques, such as UV–vis spectroscopy and ML, such a system could enable real-time monitoring of the optical properties of plasmonic nanoparticles, supporting efficient and scalable SERS substrate production.^65,109^ Automation and AI-driven quality control minimize human error and ensure the consistent fabrication of SERS-active materials, improving reproducibility in downstream SERS experiments. While robotic systems may incur higher upfront costs, they offer long-term value, especially in large-scale and high-throughput applications. First, robots are capable of handling large volumes of samples and conducting high-throughput experiments, while microfluidic systems are better suited for small-scale experiments and face challenges in scaling up. Second, robotic systems are highly adaptable and can be reprogrammed to perform a wide range of tasks across multiple chemical instruments, including sample preparation, synthesis, and data analysis. This versatility enables a wider range of experiments than microfluidics, which are typically restricted to specific workflows. Moreover, AI-powered robotic systems can analyze data in real-time and dynamically adjust experimental conditions, continuously optimizing results. This adaptability is crucial when handling unknown experiments and reactions, enabling robots to respond to unforeseen challenges. Overall, these high-throughput strategies accelerate SERS data collection, with AI-driven robotic systems providing scalability and flexibility for complex, high-throughput workflows, while multichannel microfluidic systems remain ideal for precise, small-scale experiments.

Automation in SERS, while offering great potential for increasing throughput and efficiency, still faces several significant challenges that must be addressed to unlock its full capabilities. For instance, although hardware improvements, such as enhancing spectrometer sensitivity, can reduce noise at the source, data quality remains a major challenge in high-throughput experiments. This is particularly true when dealing with complex mixtures or low-concentration analytes prone to noise, redundancy, and other inconsistencies. To improve the signal-to-noise ratio and ensure data reliability, advanced preprocessing methods such as noise-reduction algorithms, ML-based noise filtering, statistical outlier detection, and adaptive signal processing play critical roles in enhancing data quality.^58,62,100^ Additionally, incorporating replication and cross-validation strategies further enhances consistency and robustness across SERS data sets, improving the reliability across diverse experimental conditions.^60,110^ Furthermore, the lack of universally accepted reference materials and calibration standards for SERS remains a key obstacle in achieving data standardization, hindering the comparison and replication of results across studies and SERS platforms. Therefore, developing widely recognized and standardized references would enhance the consistency and utility of SERS data generated by high-throughput platforms, fostering broader application of SERS in various fields.^58,62,100^

As we look ahead, we must not forget that the vast body of existing data in the SERS literature remains an untapped goldmine for advancing SERS and cheminformatics. With the recent progress in natural language processing (NLP), specifically large language models (LLMs), researchers now have powerful tools to automatically mine published spectral databases, extract valuable insights, and identify previously overlooked patterns to accelerate the discovery process.^111,112^ For example, domain-specific model, such as MatSciBERT, a materials-aware language model trained on a large corpus of peer-reviewed materials science publications, have demonstrated success in tasks such as entity recognition, relation classification, and abstract classification (Figure 5D).^107^ This shows how LLMs can accelerate the materials discovery and information extraction from scientific texts. Building on this promise, we envision developing or fine-tuning an expert LLM tailored to the SERS domain. Such a model could automatically extract SERS spectra and other critical experimental information, such as spectrometer setups, substrates, target molecules, and methodologies, from the published literature. This helps to streamline the compilation and data mining of large SERS data sets from the literature, to enrich the centralized SERS chemical space. Beyond data mining, it would also extract meaningful spectral patterns from historical SERS spectra, offering new insights into previously underexplored molecular structures or interactions. Nevertheless, developing LLMs for SERS requires domain-specific pretraining, as general-purpose models lack the specialized knowledge needed to interpret spectroscopic data accurately. Fine-tuning on spectral-text corpora is essential, yet this demands access to high-quality, well-annotated data sets that include experimental metadata, spectral features, and analyte information, resources that are often limited or inconsistently reported.^113,114^ Furthermore, the data volume requirements for such tasks are substantial, particularly given the need to handle multimodal inputs including spectra, chemical structures, and textual descriptions.^17,111,115^ Additionally, the computational costs of training and deploying these models are significant.^111,115^ To evaluate the actionability of this strategy, we draw inspiration from previous use cases in adjacent fields such as automated spectral annotation, hypothesis generation, and spectral extraction from literature using LLM-based tools.^75,107,116–119^ While the prospect of using LLMs for SERS data mining is enticing, their application in this field comes with several challenges. First, ethical concerns, such as data privacy and potential biases in training data sets, must be carefully considered and addressed, particularly when dealing with proprietary or sensitive spectral data. Additionally, LLMS must be used transparently, with their outputs thoroughly validated to prevent the perpetuation of biases, which could lead to skewed or incomplete conclusions. Moreover, researchers must remain cautious of the risks of overreliance on such AI models, as this could undermine the critical thinking and domain-specific expertise required for rigorous scientific inquiry. Ensuring that LLMs complement, rather than replace, traditional research methodologies is key to maintaining the integrity of the research process. Collectively, our proposed two-pronged approach combining (1) advanced technologies (e.g., microfluidics, robotics) for high-throughput data generation and (2) AI and LLMs for literature mining holds immense potential to transform SERS data generation and analysis. This two-pronged approach accelerates discovery, improves reproducibility, and supports the rapid development of a robust and scalable SERS chemical space.

## 6 Conclusions and Future Outlook

Integrating SERS with cheminformatics has immense potential to revolutionize chemical analysis by enhancing spectral interpretation, predictive modeling, and data-driven discovery. Cheminformatics tools are crucial in organizing and labeling SERS spectra within centralized SERS databases, linking spectra to known chemical compounds, and enabling rapid identification and characterization of unknown substances. Computational techniques, including molecular modeling and QSAR, further enrich SERS data sets by predicting nanoparticle properties and correlating spectral features with chemical behaviors. When SERS is integrated with ML and well-curated high-quality spectral databases, SERS enables real-time high-throughput analysis, driving advancements in areas including catalysis, wearable biomedical devices, and environmental monitoring. As automation and LLM-driven data mining advance, the expanding SERS chemical space will drive discoveries, establishing SERS as a cornerstone of modern analytical chemistry. With a concerted interdisciplinary effort, the synergy between SERS and cheminformatics will continue to reshape chemical analysis, opening exciting possibilities for the future.

### 6.1 Federated Learning and Context-Aware SERS Databases

The future of a centralized and interoperable SERS database could incorporate a federated learning approach, where research laboratories contribute to the database while maintaining data privacy and security. In a federated learning system, multiple participating research laboratories could collaboratively train ML models without having to share their raw, unpublished SERS data externally. Instead, each lab would keep its data sets local and confidential before publication, training models on their secure systems. Only model updates (such as gradients or model weights) would be communicated to a central server, where they would be aggregated to update a shared global model. By enabling federated model training, each entity can enhance ML models, allowing faster updates and a more robust, real-time adaptation of the SERS database. Furthermore, advanced metadata structures could be integrated to create context-aware SERS databases that link spectral data to chemical compounds and provide contextual information about environmental factors (e.g., temperature, humidity). This would enable researchers to search for SERS spectra under specific conditions, facilitating more accurate predictions and deeper insights for specialized applications such as point-of-care diagnostics or on-site environmental monitoring.

### 6.2 Hybrid ML and Quantum Simulations

Future models could integrate ML with quantum mechanical simulations, such as quantum machine learning (QML), to predict SERS enhancement factors and optimize nanoparticle–substrate interactions for specific analytes.^96,120–124^ By combining ML’s data-driven capabilities with the precision of quantum mechanical simulations, these hybrid models would offer detailed insights into the mechanisms driving SERS enhancement. Current hybrid quantum-classical approaches for spectral prediction combine quantum computing’s potential for modeling molecular systems with classical ML to enhance accuracy and efficiency. Examples include quantum kernel methods applied to molecular property prediction, where quantum circuits encode structural information for improved classification or regression performance.^72,96,120–124^ However, these approaches face limitations due to quantum hardware noise, limited qubit counts, and scalability challenges that restrict their practical use.^96,120,121^ Looking forward, we propose benchmarking strategies that compare quantum and classical models across relevant SERS tasks, using standardized data sets and performance metrics. Notably, QML may offer distinct advantages in high-dimensional feature reduction and small-data regimes common in SERS due to experimental constraints.^96,120^ This includes information about how nanoparticles and substrates interact with target analytes. These models not only predict ideal molecular structures for SERS enhancement but also consider dynamic experimental conditions such as variations in laser intensity, temperature, and environmental factors, which influence SERS signal intensity. This would enable more robust, adaptable predictions by providing a deeper understanding of how enhancement factors fluctuate across different scenarios.^96,120,122,124^ Moreover, it could also enable real-time optimization of experimental setups, ensuring consistent, reproducible results even under varying conditions, and advancing the precision and versatility of SERS as an analytical tool.^96,121–123^ Furthermore, combining QML with computational chemistry could enable the design of novel plasmonic materials tailored to enhance SERS signals for specific molecular classes.^96,120–124^ AI-driven molecular design would enable rapid, *in silico* prototyping and testing of new materials, optimizing surface chemistry at the nano–bio interface for targeted applications in biomedical or environmental sensing.

### 6.3 Real-Time Spectral Feedback for Adaptive Experimental Control

ML models could enable real-time feedback loops, allowing the system to dynamically adjust experimental parameters such as laser power, integration time, and sample orientation during the SERS experiment based on continuous spectral analysis. This adaptive approach would enable ongoing optimization of the SERS experiment, especially in dynamic or unknown systems, where traditional static settings are inadequate. By continuously monitoring the spectral data, ML algorithms can identify subtle trends or variations that may require immediate adjustment. This ensures that the experiment remains aligned with optimal conditions throughout, particularly for complex or unpredictable samples, where traditional fixed parameters may result in suboptimal data quality. Looking ahead, as SERS technology evolves, data sets from different spectrometers or experimental platforms may vary due to hardware or experimental setup differences across laboratories. Transfer learning could enable the application of models trained on one set of equipment to another, allowing seamless integration and analysis across multiple platforms without requiring a large re-collection of data. By reusing learned spectral features and fine-tuning only for new instrument-specific variations, transfer learning reduces the need for extensive re-collection, accelerates deployment across different platforms, and ensures more consistent analysis. This approach fosters cross-platform compatibility, allowing researchers to integrate models seamlessly across diverse experimental setups without sacrificing performance. In the future, one could imagine a universal SERS model hub, where pretrained models are publicly available and users can download and adapt them to their specific experimental setups with minimal effort. This would substantially lower the technical barrier for applying ML to new SERS studies and applications in diagnostics, materials characterization, and chemical detection.

### 6.4 High-Throughput SERS-Driven Cheminformatics

The automation of SERS data acquisition, combined with cheminformatics tools, could facilitate the rapid exploration of chemical space, focusing on discovering new chemicals, reaction intermediates, and complex molecular interactions. Specifically, AI-driven robotics could automatically screen vast compound libraries, analyzing their SERS spectra to identify new molecular fingerprints and broaden the SERS chemical space. AI-powered autonomous experimentation could revolutionize the synthesis and testing of new SERS-active materials by automating the entire process from material design to validation. This will likely result in a much faster pathway to developing highly sensitive SERS substrates specifically tailored for challenging applications, such as single-molecule detection in medical diagnostics. With AI at the helm, the system could systematically explore and test various nanoparticle compositions, surface modifications, and substrate structures in an iterative process to optimize for properties, including signal enhancement, stability, and specificity. By harnessing large data sets and predictive algorithms, AI systems could rapidly pinpoint promising material combinations for targeted applications. This approach goes beyond traditional experimental design by incorporating inverse design, where AI predicts, synthesizes, and tests optimal materials in real-time. For instance, the ability to continuously iterate on material synthesis in an autonomous system would significantly accelerate the discovery and deployment of novel SERS-active materials. It also enables fine-tuning of substrate properties to the specific needs of each application rapidly and unlocks new possibilities in diagnostics and sensing.

Ongoing efforts aligned with our framework, including public and commercial SERS and Raman spectral databases,^22,69,125–130^ as well as open-source cheminformatics tools such as RDKit and PySpark,^131–133^ and recent advances in SERS–ML integration demonstrating proof-of-concept workflows for automated analyte identification and spectral interpretation,^4–6,22,34^ reinforces the feasibility of our vision. Short-term milestones include curating metadata-rich data sets and standardizing spectral formats, while mid-term goals include forming collaborative consortia, establishing data-sharing protocols, sharing molecular modeling insights, and developing cloud-based infrastructure for the centralized database. Collectively, these steps lay the foundation for cheminformatics-driven SERS research that extends beyond ML to fully leverage tools such as molecular modeling and database management, ultimately pushing the limits of SERS.

## Supplementary Material



## Notes

Transparent Peer Review report available (PDF)

## References

[1] Langer J., Jimenez de Aberasturi D., Aizpurua J., Alvarez-Puebla R. A., Auguié B., Baumberg J. J., Bazan G. C., Bell S. E. J., Boisen A., Brolo A. G. (2020). Present and Future of
Surface-Enhanced Raman Scattering. ACS Nano.

[2] Itoh T., Procházka M., Dong Z.-C., Ji W., Yamamoto Y. S., Zhang Y., Ozaki Y. (2023). Toward a New Era of SERS and TERS
at the Nanometer Scale: From Fundamentals to Innovative Applications. Chem. Rev..

[3] Chaudhry I., Hu G., Ye H., Jensen L. (2024). Toward Modeling the Complexity of
the Chemical Mechanism in SERS. ACS Nano.

[4] Tan E. X., Leong S. X., Liew W. A., Phang I. Y., Ng J. Y., Tan N. S., Lee Y. H., Ling X. Y. (2024). Forward-predictive
SERS-based chemical taxonomy for untargeted structural elucidation
of epimeric cerebrosides. Nat. Commun..

[5] Nguyen L. B. T., Tan E. X., Leong S. X., Koh C. S. L., Madhumita M., Phang I. Y., Ling X. Y. (2024). Harnessing
Cooperative Multivalency
in Thioguanine for Surface-Enhanced Raman Scattering (SERS)-Based
Differentiation of Polyfunctional Analytes Differing by a Single Functional
Group. Angew. Chem., Int. Ed..

[6] Leong S. X., Tan E. X., Han X., Luhung I., Aung N. W., Nguyen L. B. T., Tan S. Y., Li H., Phang I. Y., Schuster S. (2023). Surface-enhanced Raman scattering-based
surface chemotaxonomy:
combining bacteria extracellular matrices and machine learning for
rapid and universal species identification. ACS Nano.

[7] Leong S. X., Kao Y. C., Han X., Poh Z. W., Chen J. R. T., Tan E. X., Leong Y. X., Lee Y. H., Teo W. X., Yip G. W. (2023). Achieving Molecular Recognition of Structural Analogues
in Surface-Enhanced Raman Spectroscopy: Inducing Charge and Geometry
Complementarity to Mimic Molecular Docking. Angew. Chem., Int. Ed..

[8] Cialla-May D., Bonifacio A., Bocklitz T., Markin A., Markina N., Fornasaro S., Dwivedi A., Dib T., Farnesi E., Liu C. (2024). Biomedical SERS - the current state and future trends. Chem. Soc. Rev..

[9] Zheng X., Ye Z., Akmal Z., He C., Zhang J., Wang L. (2024). Recent progress
in SERS monitoring of photocatalytic reactions. Chem. Soc. Rev..

[10] Khatib M., Haick H. (2022). Sensors for Volatile Organic Compounds. ACS
Nano.

[11] Xie L., Gong K., Liu Y., Zhang L. (2023). Strategies and Challenges
of Identifying Nanoplastics in Environment by Surface-Enhanced Raman
Spectroscopy. Environ. Sci. Technol..

[12] Lin L. L., Alvarez-Puebla R., Liz-Marzán L.
M., Trau M., Wang J., Fabris L., Wang X., Liu G., Xu S., Han X. X. (2025). Surface-Enhanced Raman Spectroscopy for Biomedical
Applications: Recent Advances and Future Challenges. ACS Appl. Mater. Interfaces.

[13] Guselnikova O., Trelin A., Kang Y., Postnikov P., Kobashi M., Suzuki A., Shrestha L. K., Henzie J., Yamauchi Y. (2024). Pretreatment-free SERS sensing of
microplastics using
a self-attention-based neural network on hierarchically porous Ag
foams. Nat. Commun..

[14] Yang G., Xiao H., Gao H., Zhang B., Hu W., Chen C., Qiao Q., Zhang G., Feng S., Liu D. (2024). Repairing
Noise-Contaminated Low-Frequency Vibrational
Spectra with an Attention U-Net. J. Am. Chem.
Soc..

[15] Luo S.-h., Wang W.-l., Zhou Z.-f., Xie Y., Ren B., Liu G.-k., Tian Z.-q. (2022). Visualization of a Machine Learning
Framework toward Highly Sensitive Qualitative Analysis by SERS. Anal. Chem..

[16] Phan-Quang G. C., Wee E. H. Z., Yang F., Lee H. K., Phang I. Y., Feng X., Alvarez-Puebla R. A., Ling X. Y. (2017). Online Flowing Colloidosomes
for Sequential Multi-analyte High-Throughput SERS Analysis. Angew. Chem..

[17] Tan E. X., Zhong Q.-Z., Ting
Chen J. R., Leong Y. X., Leon G. K., Tran C. T., Phang I. Y., Ling X. Y. (2024). Surface-Enhanced
Raman Scattering-Based Multimodal Techniques: Advances and Perspectives. ACS Nano.

[18] Liebel M., Pazos-Perez N., van Hulst N. F., Alvarez-Puebla R. A. (2020). Surface-enhanced
Raman scattering holography. Nat. Nanotechnol..

[19] Zong C., Cheng R., Chen F., Lin P., Zhang M., Chen Z., Li C., Yang C., Cheng J.-X. (2022). Wide-field
surface-enhanced coherent anti-Stokes Raman scattering microscopy. Acs Photonics.

[20] Wang M., Zhang C., Yan S., Chen T., Fang H., Yuan X. (2021). Wide-field super-resolved
raman imaging of carbon materials. ACS Photonics.

[21] Yang W., Knorr F., Popp J., Schie I. W. (2020). Development and
evaluation of a hand-held fiber-optic Raman probe with an integrated
autofocus unit. Opt. Express.

[22] Chen J. R. T., Tan E. X., Tang J., Leong S. X., Hue S. K. X., Pun C. S., Phang I. Y., Ling X. Y. (2025). Machine Learning-Based
SERS Chemical Space for Two-Way Prediction of Structures and Spectra
of Untrained Molecules. J. Am. Chem. Soc..

[23] Yi J., You E.-M., Liu G.-K., Tian Z.-Q. (2024). AI-nano-driven surface-enhanced
Raman spectroscopy for marketable technologies. Nature Nanotechnol..

[24] Simas M. V., Davis G. A., Hati S., Pu J., Goodpaster J. V., Sardar R. (2025). Anisotropically Shaped
Plasmonic WO3-x Nanostructure-Driven
Ultrasensitive SERS Detection and Machine Learning-Based Differentiation
of Nitro-Explosives. ACS Appl. Mater. Interfaces.

[25] Ding Z., Wang C., Song X., Li N., Zheng X., Wang C., Su M., Liu H. (2023). Strong π-Metal
Interaction Enables Liquid Interfacial Nanoarray-Molecule Co-assembly
for Raman Sensing of Ultratrace Fentanyl Doped in Heroin, Ketamine,
Morphine, and Real Urine. ACS Appl. Mater. Interfaces.

[26] Simas M. V., Olaniyan P. O., Hati S., Davis G. A., Anspach G., Goodpaster J. V., Manicke N. E., Sardar R. (2023). Superhydrophobic
Surface Modification of Polymer Microneedles Enables Fabrication of
Multimodal Surface-Enhanced Raman Spectroscopy and Mass Spectrometry
Substrates for Synthetic Drug Detection in Blood Plasma. ACS Appl. Mater. Interfaces.

[27] Tang J.-W., Mou J.-Y., Chen J., Yuan Q., Wen X.-R., Liu Q.-H., Liu Z., Wang L. (2025). Discrimination of Benign
and Malignant Thyroid Nodules through Comparative Analyses of Human
Saliva Samples via Metabolomics and Deep-Learning-Guided Label-free
SERS. ACS Appl. Mater. Interfaces.

[28] Pelton J. M., Hochuli J. E., Sadecki P. W., Katoh T., Suga H., Hicks L. M., Muratov E. N., Tropsha A., Bowers A. A. (2024). Cheminformatics-Guided
Cell-Free Exploration of Peptide Natural Products. J. Am. Chem. Soc..

[29] Keith J. A., Vassilev-Galindo V., Cheng B., Chmiela S., Gastegger M., Müller K.-R., Tkatchenko A. (2021). Combining Machine Learning and Computational
Chemistry for Predictive Insights Into Chemical Systems. Chem. Rev..

[30] Lee M.-L., Farag S., Del Cid J. S., Bashore C., Hallenbeck K. K., Gobbi A., Cunningham C. N. (2023). Identification
of Macrocyclic Peptide
Families from Combinatorial Libraries Containing Noncanonical Amino
Acids Using Cheminformatics and Bioinformatics Inspired Clustering. ACS Chem. Biol..

[31] Bajomo M. M., Ju Y., Zhou J., Elefterescu S., Farr C., Zhao Y., Neumann O., Nordlander P., Patel A., Halas N. J. (2022). Computational
chromatography: A machine learning strategy for demixing individual
chemical components in complex mixtures. Proc.
Natl. Acad. Sci. U. S. A..

[32] Zou Z., Zhang Y., Liang L., Wei M., Leng J., Jiang J., Luo Y., Hu W. (2023). A deep learning
model
for predicting selected organic molecular spectra. Nature Computational Science.

[33] Wu S., Zhang Y., He C., Luo Z., Chen Z., Ye J. (2024). Self-Supervised Learning for Generic
Raman Spectrum Denoising. Anal. Chem..

[34] Leong S. X., Leong Y. X., Tan E. X., Sim H. Y. F., Koh C. S. L., Lee Y. H., Chong C., Ng L. S., Chen J. R. T., Pang D. W. C. (2022). Noninvasive and point-of-care surface-enhanced Raman
scattering (SERS)-based breathalyzer for mass screening of coronavirus
disease 2019 (COVID-19) under 5 min. ACS Nano.

[35] Kao Y.-C., Han X., Lee Y. H., Lee H. K., Phan-Quang G. C., Lay C. L., Sim H. Y. F., Phua V. J. X., Ng L. S., Ku C. W. (2020). Multiplex surface-enhanced Raman scattering identification and quantification
of urine metabolites in patient samples within 30 min. ACS Nano.

[36] Ehrentreich F., Sümmchen L. (2001). Spike removal
and denoising of Raman spectra by wavelet
transform methods. Analytical chemistry.

[37] Ju Y., Neumann O., Bajomo M., Zhao Y., Nordlander P., Halas N. J., Patel A. (2023). Identifying
Surface-Enhanced Raman
Spectra with a Raman Library Using Machine Learning. ACS Nano.

[38] Garg A., Nam W., Wang W., Vikesland P., Zhou W. (2023). In Situ Spatiotemporal
SERS Measurements and Multivariate Analysis of Virally Infected Bacterial
Biofilms Using Nanolaminated Plasmonic Crystals. ACS Sensors.

[39] Shin H., Choi B. H., Shim O., Kim J., Park Y., Cho S. K., Kim H. K., Choi Y. (2023). Single test-based
diagnosis
of multiple cancer types using Exosome-SERS-AI for early stage cancers. Nat. Commun..

[40] Lee W., Kang B.-H., Yang H., Park M., Kwak J. H., Chung T., Jeong Y., Kim B. K., Jeong K.-H. (2021). Spread
spectrum SERS allows label-free detection of attomolar neurotransmitters. Nat. Commun..

[41] Rodriguez-Nieves A. L., Taylor M. L., Wilson R., Eldridge B. K., Nawalage S., Annamer A., Miller H. G., Alle M. R., Gomrok S., Zhang D. (2024). Multiplexed Surface Protein Detection
and Cancer Classification Using
Gap-Enhanced Magnetic-Plasmonic Core-Shell Raman Nanotags and Machine
Learning Algorithm. ACS Appl. Mater. Interfaces.

[42] Das S., Saxena K., Tinguely J.-C., Pal A., Wickramasinghe N. L., Khezri A., Dubey V., Ahmad A., Perumal V., Ahmad R. (2023). SERS nanowire chip and machine learning-enabled classification of
wild-type and antibiotic-resistant bacteria at species and strain
levels. ACS Appl. Mater. Interfaces.

[43] Jiang H., Zhang Y., Zhang L., Liu L., Wang H., Wang Y., Chen M. (2025). Comprehensive Serum Analysis via
an AI-Assisted Magnetically Driven SERS Platform for the Diagnosis
and Etiological Differentiation of Childhood Epilepsy. ACS Appl. Mater. Interfaces.

[44] Leong S. X., Koh C. S. L., Sim H. Y. F., Lee Y. H., Han X., Phan-Quang G. C., Ling X. Y. (2021). Enantiospecific Molecular Fingerprinting
Using Potential-Modulated Surface-Enhanced Raman Scattering to Achieve
Label-Free Chiral Differentiation. ACS Nano.

[45] Ze H., Yang Z.-L., Li M.-L., Zhang X.-G., A Y.-L., Zheng Q.-N., Wang Y.-H., Tian J.-H., Zhang Y.-J., Li J.-F. (2024). In Situ Probing
the Structure Change and Interaction of Interfacial
Water and Hydroxyl Intermediates on Ni­(OH)­2 Surface over Water Splitting. J. Am. Chem. Soc..

[46] Reza K. K., Dey S., Wuethrich A., Jing W., Behren A., Antaw F., Wang Y., Sina A. A. I., Trau M. (2021). In Situ Single Cell
Proteomics Reveals Circulating Tumor Cell Heterogeneity during Treatment. ACS Nano.

[47] Bi X., Qian X., Xue B., Zhang M., Liu S., Chen H., Jin C., Tang H., Ye J. (2025). Molecule-resolvable
SERSome for metabolic profiling. Chem..

[48] Zhao F., Zheng Y., Zhao Z., Wang W., Xu T., Xue X., Fu W., Ling Y., Shi J., Zhang Z. (2024). Re-understanding
of SERS for General and Standardized Quantitative Analysis. Nano Lett..

[49] Cai W., Xie X., Yang Z., Guo X. (2025). Stereochemistry at the Single-Molecule
Level: From Monitoring to Regulation. Angew.
Chem., Int. Ed..

[50] Luo X., Zhao X., Wallace G. Q., Brunet M.-H. n., Wilkinson K. J., Wu P., Cai C., Bazuin C. G., Masson J.-F. (2021). Multiplexed SERS
detection of microcystins with aptamer-driven core-satellite assemblies. ACS Appl. Mater. Interfaces.

[51] Huang J.-A., Mousavi M. Z., Zhao Y., Hubarevich A., Omeis F., Giovannini G., Schütte M., Garoli D., De Angelis F. (2019). SERS discrimination
of single DNA
bases in single oligonucleotides by electro-plasmonic trapping. Nat. Commun..

[52] Siddhanta S., Wróbel M. S., Barman I. (2016). Integration of protein
tethering
in a rapid and label-free SERS screening platform for drugs of abuse. Chem. Commun..

[53] Ma H., Yan S., Lu X., Bao Y.-F., Liu J., Liao L., Dai K., Cao M., Zhao X., Yan H. (2023). Rapidly determining
the 3D structure of proteins by surface-enhanced Raman spectroscopy. Science Advances.

[54] Zhang Y., Chang K., Ogunlade B., Herndon L., Tadesse L. F., Kirane A. R., Dionne J. A. (2024). From genotype
to phenotype: Raman
spectroscopy and machine learning for label-free single-cell analysis. ACS Nano.

[55] Boudries R., Williams H., Paquereau-Gaboreau S., Bashir S., Hojjat Jodaylami M., Chisanga M., Trudeau L.-É., Masson J.-F. (2024). Surface-Enhanced
Raman Scattering Nanosensing and Imaging in Neuroscience. ACS Nano.

[56] Guo S., Popp J., Bocklitz T. (2021). Chemometric
analysis in Raman spectroscopy
from experimental design to machine learning-based modeling. Nature protocols.

[57] Guo S., Beleites C., Neugebauer U., Abalde-Cela S., Afseth N. K., Alsamad F., Anand S., Araujo-Andrade C., Askrabic S., Avci E. (2020). Comparability of Raman
spectroscopic
configurations: a large scale cross-laboratory study. Analytical chemistry.

[58] Jiang X., Yang M., Meng Y., Jiang W., Zhan J. (2013). Cysteamine-modified
silver nanoparticle aggregates for quantitative SERS sensing of pentachlorophenol
with a portable Raman spectrometer. ACS Appl.
Mater. Interfaces.

[59] Wolf S., Domes R., Merian A., Domes C., Frosch T. (2022). Parallelized
Raman difference spectroscopy for the investigation of chemical interactions. Anal. Chem..

[60] Yang G., Xiao H., Gao H., Zhang B., Hu W., Chen C., Qiao Q., Zhang G., Feng S., Liu D. (2024). Repairing Noise-Contaminated
Low-Frequency Vibrational Spectra with
an Attention U-Net. J. Am. Chem. Soc..

[61] Kazemzadeh M., Martinez-Calderon M., Xu W., Chamley L. W., Hisey C. L., Broderick N. G. (2022). Cascaded deep convolutional neural networks as improved
methods of preprocessing raman spectroscopy data. Anal. Chem..

[62] Cerjan B., Yang X., Nordlander P., Halas N. J. (2016). Asymmetric aluminum
antennas for self-calibrating surface-enhanced infrared absorption
spectroscopy. ACS Photonics.

[63] Heraud P., Wood B. R., Beardall J., McNaughton D. (2006). Effects of
pre-processing of Raman spectra on in vivo classification of nutrient
status of microalgal cells. Journal of Chemometrics:
A Journal of the Chemometrics Society.

[64] Luo S.-h., Zhao X.-j., Cao M.-f., Xu J., Wang W.-l., Lu X.-y., Huang Q.-t., Yue X.-x., Liu G.-k., Yang L. (2024). Signal2signal: Pushing the Spatiotemporal
Resolution to the Limit
by Single Chemical Hyperspectral Imaging. Anal.
Chem..

[65] Tan E. X., Tang J., Leong Y. X., Phang I. Y., Lee Y. H., Pun C. S., Ling X. Y. (2024). Creating 3D Nanoparticle
Structural
Space via Data Augmentation to Bidirectionally Predict Nanoparticle
Mixture’s Purity, Size, and Shape from Extinction Spectra. Angew. Chem..

[66] Van
Dyk D. A., Meng X.-L. (2001). The art of data augmentation. Journal of Computational and Graphical Statistics.

[67] Maharana K., Mondal S., Nemade B. (2022). A review:
Data pre-processing and
data augmentation techniques. Global Transitions
Proceedings.

[68] Wigh D. S., Goodman J. M., Lapkin A. A. (2022). A review of molecular
representation
in the age of machine learning. Wiley Interdisciplinary
Reviews: Computational Molecular Science.

[69] Joung J. F., Han M., Hwang J., Jeong M., Choi D. H., Park S. (2021). Deep learning
optical spectroscopy based on experimental database: potential applications
to molecular design. JACS Au.

[70] Weininger D. (1988). SMILES, a
chemical language and information system. 1. Introduction to methodology
and encoding rules. Journal of chemical information
and computer sciences.

[71] Xu S., Li J., Cai P., Liu X., Liu B., Wang X. (2021). Self-improving
photosensitizer discovery system via bayesian search with first-principle
simulations. J. Am. Chem. Soc..

[72] Ju C.-W., Bai H., Li B., Liu R. (2021). Machine learning enables highly accurate
predictions of photophysical properties of organic fluorescent materials:
Emission wavelengths and quantum yields. J.
Chem. Inf. Model..

[73] Tetko I. V., Engkvist O., Koch U., Reymond J. L., Chen H. (2016). BIGCHEM: challenges
and opportunities for big data analysis in chemistry. Molecular informatics.

[74] Wilkinson M. D., Dumontier M., Aalbersberg I. J., Appleton G., Axton M., Baak A., Blomberg N., Boiten J.-W., da Silva
Santos L. B., Bourne P. E. (2016). The FAIR Guiding Principles for scientific
data management and stewardship. Scientific
data.

[75] Gauglitz J. M., West K. A., Bittremieux W., Williams C. L., Weldon K. C., Panitchpakdi M., Di Ottavio F., Aceves C. M., Brown E., Sikora N. C. (2022). Enhancing untargeted metabolomics using metadata-based
source annotation. Nature biotechnology.

[76] Langer J., Jimenez de Aberasturi D., Aizpurua J., Alvarez-Puebla R. A., Auguié B., Baumberg J. J., Bazan G. C., Bell S. E., Boisen A., Brolo A. G. (2020). Present and future of surface-enhanced
Raman scattering. ACS Nano.

[77] Campion A., Kambhampati P. (1998). Surface-enhanced
Raman scattering. Chem. Soc. Rev..

[78] Orio M., Pantazis D. A., Neese F. (2009). Density functional
theory. Photosynthesis research.

[79] Cohen A. J., Mori-Sánchez P., Yang W. (2012). Challenges for density functional
theory. Chem. Rev..

[80] Lin J., Ren W., Li A., Yao C., Chen T., Ma X., Wang X., Wu A. (2020). Crystal-amorphous
core-shell structure
synergistically enabling TiO2 nanoparticles’ remarkable SERS
sensitivity for cancer cell imaging. ACS Appl.
Mater. Interfaces.

[81] Fraser J. P., Postnikov P., Miliutina E., Kolska Z., Valiev R., Švorčík V., Lyutakov O., Ganin A. Y., Guselnikova O. (2020). Application
of a 2D molybdenum telluride in SERS detection
of biorelevant molecules. ACS Appl. Mater. Interfaces.

[82] Leong S. X., Koh L. K., Koh C. S. L., Phan-Quang G. C., Lee H. K., Ling X. Y. (2020). In situ differentiation of multiplex
noncovalent interactions using sers and chemometrics. ACS Appl. Mater. Interfaces.

[83] Morris G. M., Lim-Wilby M. (2008). Molecular
docking. Molecular
modeling of proteins.

[84] Fan J., Fu A., Zhang L. (2019). Progress in molecular docking. Quantitative Biology.

[85] Hollingsworth S. A., Dror R. O. (2018). Molecular dynamics simulation for
all. Neuron.

[86] Deneme I., Liman G., Can A., Demirel G., Usta H. (2021). Enabling three-dimensional
porous architectures via carbonyl functionalization and molecular-specific
organic-SERS platforms. Nat. Commun..

[87] Golubewa L., Karpicz R., Matulaitiene I., Selskis A., Rutkauskas D., Pushkarchuk A., Khlopina T., Michels D., Lyakhov D., Kulahava T. (2020). Surface-enhanced
Raman spectroscopy of organic molecules
and living cells with gold-plated black silicon. ACS Appl. Mater. Interfaces.

[88] Shermy R., Saranya N. (2025). Cloud-Based Big Data
Architecture and Infrastructure. Resilient Community
Microgrids.

[89] Jackson, K. R. ; Ramakrishnan, L. ; Muriki, K. ; Canon, S. ; Cholia, S. ; Shalf, J. ; Wasserman, H. J. ; Wright, N. J. Performance analysis of high performance computing applications on the amazon web services cloud. In 2010 IEEE second international conference on cloud computing technology and science; IEEE: 2010; pp 159–168.

[90] Hansson T., Oostenbrink C., van Gunsteren W. (2002). Molecular dynamics simulations. Curr. Opin. Struct. Biol..

[91] Ryno S. M., Ravva M. K., Chen X., Li H., Brédas J. L. (2017). Molecular
understanding of fullerene-electron donor interactions in organic
solar cells. Adv. Energy Mater..

[92] Gong W., Xie Y., Pham T. D., Shetty S., Son F. A., Idrees K. B., Chen Z., Xie H., Liu Y., Snurr R. Q. (2022). Creating
optimal pockets in a Clathrochelate-based metal-organic framework
for gas adsorption and separation: Experimental and computational
studies. J. Am. Chem. Soc..

[93] Ju Y., Neumann O., Denison S. B., Jin P., Sanchez-Alvarado A. B., Nordlander P., Senftle T. P., Alvarez P. J., Patel A., Halas N. J. (2025). In silico
machine learning-enabled detection of polycyclic
aromatic hydrocarbons from contaminated soil. Proc. Natl. Acad. Sci. U. S. A..

[94] Wu Z., Zhu M., Kang Y., Leung E. L.-H., Lei T., Shen C., Jiang D., Wang Z., Cao D., Hou T. (2021). Do we need
different machine learning algorithms for QSAR modeling? A comprehensive
assessment of 16 machine learning algorithms on 14 QSAR data sets. Briefings in bioinformatics.

[95] Pirhadi S., Shiri F., Ghasemi J. B. (2015). Multivariate
statistical analysis
methods in QSAR. Rsc Advances.

[96] Metawei, M. A. ; Said, H. ; Taher, M. ; Eldeib, H. ; Nassar, S. M. Survey on hybrid classical-quantum machine learning models. In 2020 International Conference on Communications, Computing, Cybersecurity, and Informatics (CCCI); IEEE: 2020; pp 1–6.

[97] Hegde A., Hajikhani M., Snyder J., Cheng J., Lin M. (2025). Leveraging
SERS and Transformer Models for Simultaneous Detection of Multiple
Pesticides in Fresh Produce. ACS Appl. Mater.
Interfaces.

[98] Hwang C. S., Lee S., Lee S., Kim H., Kang T., Lee D., Jeong K.-H. (2022). Highly adsorptive Au-TiO2 nanocomposites for the SERS
face mask allow the machine-learning-based quantitative assay of SARS-CoV-2
in artificial breath aerosols. ACS Appl. Mater.
Interfaces.

[99] Tseng Y.-M., Chen K.-L., Chao P.-H., Han Y.-Y., Huang N.-T. (2023). Deep learning-assisted
surface-enhanced raman scattering for rapid bacterial identification. ACS Appl. Mater. Interfaces.

[100] Wang X., Jiang S., Hu W., Ye S., Wang T., Wu F., Yang L., Li X., Zhang G., Chen X. (2022). Quantitatively determining surface-adsorbate
properties from vibrational spectroscopy with interpretable machine
learning. J. Am. Chem. Soc..

[101] Chen Z., Wang W., Tian H., Yu W., Niu Y., Zheng X., Liu S., Wang L., Huang Y. (2024). Wearable intelligent
sweat platform for SERS-AI diagnosis of gout. Lab Chip.

[102] Qu C., Fang H., Yu F., Chen J., Su M., Liu H. (2024). Artificial nose of scalable plasmonic array gas sensor for Multi-Dimensional
SERS recognition of volatile organic compounds. Chemical Engineering Journal.

[103] Mogera U., Guo H., Namkoong M., Rahman M. S., Nguyen T., Tian L. (2022). Wearable plasmonic
paper-based microfluidics
for continuous sweat analysis. Science advances.

[104] Saxena, S. ; Gupta, S. Practical real-time data processing and analytics: distributed computing and event processing using Apache Spark, Flink, Storm, and Kafka; Packt Publishing Ltd: 2017.

[105] Verma S., Bala A. (2021). Auto-scaling techniques for IoT-based
cloud applications: a review. Cluster Computing.

[106] Burger B., Maffettone P. M., Gusev V. V., Aitchison C. M., Bai Y., Wang X., Li X., Alston B. M., Li B., Clowes R. (2020). A mobile robotic chemist. Nature.

[107] Gupta T., Zaki M., Krishnan N. A., Mausam (2022). MatSciBERT: A materials domain language
model for text mining and information extraction. npj Computational Materials.

[108] Ayora Canada M. J., Ruiz Medina A., Frank J., Lendl B. (2002). Bead injection
for surface enhanced Raman spectroscopy: automated on-line monitoring
of substrate generation and application in quantitative analysis. Analyst.

[109] Tan E. X., Chen Y., Lee Y. H., Leong Y. X., Leong S. X., Stanley C. V., Pun C. S., Ling X. Y. (2022). Incorporating
plasmonic featurization with machine learning to achieve accurate
and bidirectional prediction of nanoparticle size and size distribution. Nanoscale Horizons.

[110] Widrow B., Glover J. R., McCool J. M., Kaunitz J., Williams C. S., Hearn R. H., Zeidler J. R., Dong J. E., Goodlin R. C. (1975). Adaptive noise cancelling: Principles
and applications. Proceedings of the IEEE.

[111] Wu, S. ; Fei, H. ; Qu, L. ; Ji, W. ; Chua, T.-S. Next-gpt: Any-to-any multimodal llm. In Forty-first International Conference on Machine Learning; 2024.

[112] Ramos M.
C., Collison C. J., White A. D. (2025). A review of large
language models and autonomous agents in chemistry. Chemical Science.

[113] Nitin, V. ; Krishna, R. ; Ray, B. Spectra: Enhancing the code translation ability of language models by generating multi-modal specifications. arXiv preprint arXiv:2405.18574; 2024.

[114] Crespo-Sanchez M., Lopez-Arevalo I., Aldana-Bobadilla E., Molina-Villegas A. (2022). A content spectral-based text representation. Journal of Intelligent & Fuzzy Systems.

[115] Hu, W. ; Xu, Y. ; Li, Y. ; Li, W. ; Chen, Z. ; Tu, Z. Bliva: A simple multimodal llm for better handling of text-rich visual questions. In Proceedings of the AAAI Conference on Artificial Intelligence; 2024; Vol. 38, pp 2256–2264.

[116] Hoffmann M. A., Nothias L.-F., Ludwig M., Fleischauer M., Gentry E. C., Witting M., Dorrestein P. C., Dührkop K., Böcker S. (2022). High-confidence structural annotation
of metabolites absent from spectral libraries. Nat. Biotechnol..

[117] Brademan D. R., Riley N. M., Kwiecien N. W., Coon J. J. (2019). Interactive
peptide spectral annotator: a versatile web-based tool for proteomic
applications. Molecular & Cellular Proteomics.

[118] Liang J., Yu X., Hong W., Cai Y. (2024). Information
extraction of UV-NIR spectral data in waste water based on Large Language
Model. Spectrochimica Acta Part A: Molecular
and Biomolecular Spectroscopy.

[119] Lavoie F. B., Braidy N., Gosselin R. (2016). Including noise characteristics
in MCR to improve mapping and component extraction from spectral images. Chemometrics and Intelligent Laboratory Systems.

[120] Yordanov Y. S., Arvidsson-Shukur D. R., Barnes C. H. (2020). Efficient quantum
circuits for quantum computational chemistry. Phys. Rev. A.

[121] Torabian, E. ; Krems, R. V. Molecular representations of quantum circuits for quantum machine learning. arXiv preprint arXiv:2503.05955; 2025.

[122] Sajjan M., Li J., Selvarajan R., Sureshbabu S. H., Kale S. S., Gupta R., Singh V., Kais S. (2022). Quantum machine learning for chemistry and physics. Chem. Soc. Rev..

[123] Ramakrishnan R., von Lilienfeld O. A. (2017). Machine
learning, quantum chemistry,
and chemical space. Reviews in computational
chemistry.

[124] Huang B., Symonds N. O., von Lilienfeld O. A. (2020). Quantum
machine learning in chemistry and materials. Handbook of Materials Modeling: Methods: Theory and Modeling.

[125] Yuan Q., Tang J.-W., Chen J., Liao Y.-W., Zhang W.-W., Wen X.-R., Liu X., Chen H.-J., Wang L. (2025). SERS-ATB: A comprehensive database
server for antibiotic SERS spectral
visualization and deep-learning identification. Environ. Pollut..

[126] Wang A., Han J., Guo L., Yu J., Zeng P. (1994). Database of standard
Raman spectra of minerals and related inorganic
crystals. Appl. Spectrosc..

[127] Shi H., Wang H., Meng X., Chen R., Zhang Y., Su Y., He Y. (2018). Setting up
a surface-enhanced Raman scattering database
for artificial-intelligence-based label-free discrimination of tumor
suppressor genes. Analytical chemistry.

[128] Sherman L. M., Petrov A. P., Karger L. F., Tetrick M. G., Dovichi N. J., Camden J. P. (2020). A surface-enhanced Raman spectroscopy
database of 63 metabolites. Talanta.

[129] De Gelder J., De Gussem K., Vandenabeele P., Moens L. (2007). Reference database of Raman spectra
of biological molecules. Journal of Raman Spectroscopy:
An International Journal for
Original Work in all Aspects of Raman Spectroscopy, Including Higher
Order Processes, and also Brillouin and Rayleigh Scattering.

[130] Candeloro P., Grande E., Raimondo R., Di Mascolo D., Gentile F., Coluccio M. L., Perozziello G., Malara N., Francardi M., Di Fabrizio E. (2013). Raman database
of amino acids solutions: A critical study of Extended Multiplicative
Signal Correction. Analyst.

[131] Lovrić M., Molero J. M., Kern R. (2019). PySpark and
RDKit:
moving towards big data in cheminformatics. Molecular informatics.

[132] Landrum G. (2013). Rdkit documentation. Release.

[133] Bento A. P., Hersey A., Félix E., Landrum G., Gaulton A., Atkinson F., Bellis L. J., De Veij M., Leach A. R. (2020). An open source chemical structure
curation pipeline using RDKit. Journal of Cheminformatics.

