# Supplementary material for: SERS Cheminformatics: Opportunities for Data-Driven Discovery and Applications
Source: ACS Cent Sci. 2025 Aug 5;11(11):2034–52. doi: 10.1021/acscentsci.5c00785 (PMC12670308; doi:10.1021/acscentsci.5c00785)
Supplement: Supplementary file 1 [file oc5c00785_si_001.pdf]

oc-2025-00785q.R1

Name: Peer Review Information for "SERS Cheminformatics: Opportunities for Data-Driven Discovery and Applications"

First Round of Reviewer Comments

Reviewer: 1

Comments to the Author

This Outlook presents a high-level, visionary view of how the powerful method of SERS and the evolving machine learning algorithms can come together to enable data-driven discovery. It should be of interest to abroad range of researchers in chemistry and related disciplines.

The work is well-structured and easy to follow.

The application of the concept of "federated learning" to SERS analysis is appealing.

Some minor comments are given below.

1. "In another study, SERS and DFT analysis demonstrated that

carbonyl functionalization within porous architectures incurred chemical enhancement on organic SERS platforms. Further DFT analysis revealed that π-extended LUMOs and differences variations in crystalline orientations of the highly crystalline nanostructured D(C7CO)-[1]benzothieno[3,2-b][1]benzothiophenes (BTBT) films contribute significantly to SERS signals."

These two sentences are difficult to follow, please consider rephrasing them.

2. The figures are low resolution, and difficult to follow. For example, Figure 4 has a lot of information, part of the notations has been lost, and images appear vertically compressed.

3. p.27, please consider rephrasing the following: "These hurdles delay understanding of insights and hinder prompt decision-making in timesensitive applications".

4. General proof-reading may be required - see, for example, p. 27 "On one work", p.27 "quantified uric acid levels in both physiological and pathological levels of 1 μM,"

Overall, a detailed, interesting, forward-looking article which will be useful for many.

Reviewer: 2

Comments to the Author

Authors claimed that they develope a SERS based omics analysis.

I think their concept is very attractive.However, theire experimental results are not appropriate and in-efficient.

To become informatics, I think that they need to show the whole structure of molecules or a target molecule structure in a mixture condition. But they can show the structure of a small functional group.

In Fig 3C and 3D, Raman signals change with respect to time is shown, but I am wondering if this is a evidence to reveal a speicific functional structure.

In Fig 4, I think all results or applications can be repeated with a plane Raman signals. I am wondering whether these are good examples to show Raman based chemoinformatics.

Reviewer: 3

Comments to the Author

The Outlook by Tan and coworkers is a comprehensive overview of how the SERS technique can be more broadly employed and used by combining physical experimentation with increasingly important automation, computation, and machine learning techniques. As expected for this type of article, the authors do an excellent job of putting the topic in context and heavily employ their perspective on the current state-of-the-art and outlook. While well written, the article is going to be especially attractive to experts and users in this area, but it's less clear how comprehensible this outlook will be to a general reader. While SERS is an oft-used technique, it may be worthwhile to include a short section in the beginning of the piece describing exactly what the technique is and what it measures, and the data produced. This would go a long way to help a general reader to understand the value and importance of this work.

Reviewer: 4

Comments to the Author

This outlook-based article describes a proposed vision and associated review for surface enhanced Raman scattering (SERS) in conjunction with cheminformatics for applications in biochemical analysis and discovery. The authors propose a framework that relies on SERS databases, molecular modeling, machine learning, and automation to further unify the use of SERS with cheminformatics. Please find below my comments:

• The introduction requires revision, with an emphasis on lacking appropriate references. Examples includes where the authors state "Moreover, recent advances in both methodology and hardware have dramatically increased the volume and complexity of SERS data generated globally." (i.e., citations are needed for specific methodology and hardware) and where the authors also state "Currently, cheminformatics is employed for tasks such as peak identification, spectral noise reduction, multivariate statistical analysis, and pattern recognition, all of which support the identification of unknown chemicals." (i.e., similarly, citations are needed).

- The authors focus heavily on molecular identification in complex mixtures throughout the manuscript. While this is certainly a relevant application, SERS can be useful for many other applications that don't involve unknown structures or complex mixtures of unknown composition. If the authors are proposing an outlook for SERS and cheminformatics generally, a broader view should be taken, including relevant literature and prospective views of the future vision in a more diverse, widespread manner.

- Regarding the four pillars outlined by the authors, the fourth pillar seems very broad, encompassing both automation of physical laboratory measurements via robotics with AI-driven data mining in a single pillar. I would propose these topics could be addressed separately.

- Looking at the list of high impact applications the authors predict advances in cheminformatics and SERS could enable (Pg 4 lines 46-54), I'm not clear on why the authors choose to focus on targeting "multiplex detection of stereoisomers and alkyl substituted analogs" via SERS, given that existing analytical methods routinely solve this challenge. Could the authors expand on why this is a high impact area where SERS is uniquely positioned to make novel impact? The associated discussion later (Pg 15) needs to be addressed, including why this is a problem SERS is particularly well-positioned to address.

- Similarly, regarding application #3 (Pg. 4 lines 46-54), with regards to the discussion of genomics and proteomics as high impact SERS applications, could the authors please expand on this more specifically? And moreover, elaborate and clarify the cases in which SERS could become a preferred analytical method? Mapping genomes or proteomes sounds like a particularly difficult challenge with SERS and it's not clear to me in what cases SERS would be a preferred method of measurement over existing techniques in these fields. There are other perhaps more obvious advancements I could envision, such as further enhancing limits of detection using the framework the authors outline, so it would be useful to provide more detail on why specifically these envisioned applications were highlighted.

- The use of molecular dynamics simulations to confirm spectral assignments certainly makes sense in some cases. In complex mixtures of unknown composition, where de novo molecular structure identification is the “holy grail,” could the authors please discuss how molecular dynamics do or do not participate in their framework? I’m not clear on how useful simulations will be when the overall composition of the mixture is unknown and identifying individual components is a challenge. Also, building on this, given the work that exists in the literature for simulated Raman spectra and associated model training of known composition, the authors should identify and present the key challenges and/or limitations associated with this current state to then build a future-looking vision that is both specific and detailed.

- The authors mention standard databases for different substrates and instruments. Given the body of work that exists in the literature to date for evaluating different substrates across diverse instrumentation, the authors should clarify what they are proposing differently? How is the field advancing relative to this body of work? Detailing a summary of current literature reports that then proposed a future proposed guideline is needed, including covering the topic of when a SERS-based methodology is applied across disparate analytical instrumentation, how can we robustly determine the similarity of the generated results and account for differences in instrumental design, calibration, and other key analytical features inherent to the instrument itself?

- The authors proposal for developing LLMs for SERS should present a more thorough foundation that includes, for instance, what are the costs associated with developing a LLM for SERS, what specific type and level of fine tuning is required, how robust is this proposed fine tuning, and other critical information that can turn this proposed vision from a more lofty vision to a realistic aspirational goal the field should move towards.

- Furthermore, the authors mention quantum machine learning (QML) and to make this section more impactful, the authors should provide a more thorough overview of the topic. The current description seems vague and only hints at the advantages of such techniques, but fails to address the current state of the field, any challenges and/or limitations with development and subsequent implementation, and what the proposal for benchmarking of methodologies either currently is or may look like in the future.

• A broader question I leave wondering is how close the field is to achieving the goals outlined by the authors in this outlook. Providing additional detail on efforts currently underway and/or pointing readers to existing databases being constructed, etc. is needed.

Author's Response to Peer Review Comments:

Formatting Needs:

We thank the editor for his guidance. In this revision, we have edited the manuscript (highlighted in yellow) to fully comply with formatting needs.

Author List: Please include the email address(es) of the corresponding author(s) on the first page of the manuscript.

We included the email addresses of the corresponding author(s) on the first page of the manuscript.

Graphics: Copyright permissions are required for any graphic that is reproduced or adapted from a source that is not published by the American Chemical Society. Many of your figures currently contain reference citations. Please make sure that all graphics reproduced or adapted from another journal have the proper permission forms and credit lines in your revision. If the document list is extensive, you may consider compressing the files and uploading a ".zip" file "for Editors only".

For more information on image permissions, see: http://pubs.acs.org/page/copyright/permissions_otherpub.html

*If your graphic is reproduced or adapted from a source that is not published by the ACS, please submit the copyright permission form obtained from the other publisher as "Other Files for Editors Only". Please ensure that the licenses provide 1) "Type of use" as "reuse in journal/magazine", "Title of targeted journal" as "Energy & Fuels", and 3) "Publisher" as "American Chemical Society". Most copyright permissions can be obtained from the publishers' websites. Please also make sure a permission statement is included in the caption, if you have not already done so, e.g., Reproduced or adapted with permission from [provide reference #]. Copyright [YEAR] [PUBLISHER].

*If you are using material that appeared in any ACS journal, and ACS owns copyright to the material, permission is NOT necessary. A simple acknowledgment is required, in this format, after inserting the appropriate information where the capitalized words appear: Reproduced from

[PROVIDE REFERENCE #]. Copyright [YEAR] American Chemical Society.

*If you are using material that is covered under an Open Access license, please provide proper attribution, e.g., Reproduced from ref XX. Available under a CC-BY XX license. Copyright XXXX Author Name(s). For information about proper attribution, please see https://wiki.creativecommons.org/wiki/Best_practices_for_attribution.

We have added the credit lines and included the copyright permission forms for all graphics that are reproduced or adapted from sources not published by the American Chemical Society in a .zip file "for Editors only". For graphics that are reproduced or adapted from the American Chemical Society, credit lines are included according to instructions.

Pull Quotes (Outlook): We encourage you to select 3 - 4 quotes from your Outlook that you would like highlighted in your paper. The quotes should be one sentence-long, unique to the Outlook and not from previously cited work. Please list your quotes at the end of the manuscript file.

We selected 4 quotes and provided them at the end of the manuscript file **on page 51**, including:

1. "We propose a conceptual framework built upon four interconnected pillars: centralized SERS databases, molecular modeling, machine learning, and AI-driven automation to accelerate cheminformatics-driven discovery."
2. "SERS and cheminformatics form a powerful synergy, whereby SERS delivers highthroughput, real-time molecular data, while cheminformatics organizes and interprets this large volume of data to drive smart data-driven chemical analysis."
3. "Our vision redefines SERS from a traditional measurement technique into a data-centric platform where autonomous experimentation, cheminformatics, and LLM-driven mining converge to expand the chemical space at unprecedented speed and precision."
4. "A centralized SERS database can serve as a key resource for cheminformatics, empowering the SERS community to harness collective insights from a cohesive and interoperable dataset and enabling reliable and universally trusted spectral interpretations."

Synopsis: ACS Central Science requires a brief synopsis. The synopsis should be no more than 200 characters (including spaces) and should reasonably correlate with the Table of Contents (TOC) graphic. The synopsis is intended to explain the importance of the article to a broader readership across the sciences. Please place your synopsis in the manuscript file after the TOC graphic and label as "Synopsis."

We included a brief synopsis and placed it after the TOC graphics **on page 52**:
Synopsis: "This Outlook envisions a data-centric transformation of SERS, integrating centralized databases, cheminformatics, and AI-driven automation to accelerate chemical discovery with unprecedented speed and precision."

TOC Graphic: Include a TOC graphic illustrating the significance of the paper. The TOC graphic should be something that is representative of your entire work. Color schemes or illustrations typically make good choices. The TOC graphic must be original and free from any copyright issues. Confirm that all text is legible. Present the TOC graphic on the last page of the manuscript by itself. Please label the TOC as "TOC Graphic". A caption describing the TOC is not needed. Please see more information/guidelines for TOC Graphics at the following link:
http://pubsapp.acs.org/paragonplus/submission/toc_abstract_graphics_guidelines.pdf?

We included a TOC graphic **on page 52** of the manuscript, labeled as "TOC Graphic".
All images are original and generated by the authors using CorelDRAW with no copyright figures.

Reviewer: 1

We thank the reviewer for his/her positive appraisal and thorough review. In this revision, we have edited the manuscript (highlighted in green) according to the reviewer's comments:

Some minor comments are given below.

1. "In another study, SERS and DFT analysis demonstrated that carbonyl functionalization within porous architectures incurred chemical enhancement on organic SERS platforms. Further DFT analysis revealed that π-extended LUMOs and differences variations in crystalline orientations of the highly crystalline nanostructured D(C7CO)-[1]benzothieno[3,2-b][1]benzothiophenes (BTBT) films contribute significantly to SERS signals."These two sentences are difficult to follow, please consider rephrasing them.

We thank the reviewer for pointing this out. We have revised the passage for clarity.
The revised version on **page 19** reads: "In a separate study, combined SERS and DFT analyses showed that incorporating carbonyl groups into porous architectures enhances the SERS response of organic platforms via a chemical enhancement mechanism. As revealed by DFT modelling, this enhancement was attributed to π-extended LUMOs and to variations in crystalline orientation within highly ordered nanostructured films of D(C_7CO)-[1]benzothieno[3,2-*b*][1]benzothiophenes (BTBT)."

2. The figures are low resolution, and difficult to follow. For example, Figure 4 has a lot of information, part of the notations has been lost, and images appear vertically compressed.

We appreciate this valuable feedback. we have replaced figures 4C and D with high-resolution versions and ensured that the aspect ratios are preserved to avoid visual compression. Additionally, we clarified the notations and verified legibility across various display formats (all text > 5pt) for visual clarity.
These changes are reflected in the revised figure 4 on **page 26**.

3. p.27, please consider rephrasing the following: "These hurdles delay understanding of insights and hinder prompt decision-making in timesensitive applications".

Thank you for the suggestion. We have revised the sentence to improve flow and readability. The new version on **page 27** reads: "These hurdles slow the extraction of

insights and impede timely decision-making in time-sensitive applications where rapid responses are critical."

4. General proof-reading may be required - see, for example, p. 27 "On one work", p.27 "quantified uric acid levels in both physiological and pathological levels of 1 μM,"

We appreciate your attention to these errors. We have carefully proofread the manuscript and corrected the identified issues according to your instructions:

- On **page 27** the phrase "On one work," has been revised to "In one study,"
- On **page 27**, the sentence "...quantified uric acid levels in both physiological and pathological levels of 1 μM," has been revised to "...quantified uric acid at both physiological and pathological levels of 1 μM"

We have also performed a comprehensive language review to correct similar issues throughout the manuscript.

Reviewer: 2

Comments:
We thank the reviewer for his/her constructive review. In this revision, we have answered the reviewer's comments:

Authors claimed that they developed a SERS based omics analysis. I think their concept is very attractive. However, their experimental results are not appropriate and in-efficient.

We thank the reviewer for his/her thoughtful evaluation and are encouraged by their interest in the proposed SERS-based cheminformatics framework. We respectfully clarify that our primary objective in this Outlook is not to claim full realization of a complete omics-scale SERS informatics system, but rather to articulate a conceptual roadmap toward this goal.

Importantly, this Outlook is a perspective-style article rather than an original research article. All the experimental examples are extracted from recent literature to illustrate the foundational principles and emerging capabilities that support this vision, particularly in identifying functional groups, detecting dynamic chemical changes, and capturing molecular-level heterogeneity in complex environments.

To become informatics, I think that they need to show the whole structure of molecules or a target molecule structure in a mixture condition. But they can show the structure of a small functional group.

Regarding the reviewer's concern that identification of only small functional groups may not constitute true cheminformatics: we agree that full molecular structure elucidation in unknown mixtures remains a challenge. However, we contend that the ability to detect and differentiate functional motifs in situ, especially in dynamic or heterogeneous biochemical contexts, represents a critical and necessary step toward scalable molecular understanding. SERS offers unique advantages in this regard by enhancing subtle vibrational signatures that are often masked in conventional Raman spectra. Functional group-level resolution can provide insight into chemical reactivity, binding modes, or molecular recognition, relevant to omics-scale analyses.

In Fig 3C and 3D, Raman signals change with respect to time is shown, but I am wondering if this is a evidence to reveal a specific functional structure.

We thank the reviewer for this question. Figures 3C and 3D demonstrate how molecular docking and molecular dynamics simulations can model evolving molecular interactions that, in turn, influence SERS spectra before and after binding events. Temporal spectral changes can be tracked by SERS when they occur on accessible timescales, such as microseconds for molecular conformational changes. While these simulations do not provide definitive structural elucidation, they capture shifts in specific spectral regions associated with dynamic interaction states, enabling differentiation of analytes ranging from small drug metabolites to whole bacteria.

In Fig 4, I think all results or applications can be repeated with a plane Raman signals. I am wondering whether these are good examples to show Raman based chemoinformatics.

We appreciate the reviewer's observation. In Figure 4, while many of the analyses shown could theoretically be conducted with conventional Raman, SERS offers enhanced sensitivity, enabling detection of small gaseous analytes with small Raman scattering efficiencies at lower concentrations and in complex sample matrices. These are exemplified in Figures 4 A-D, which show targeted SERS detection of specific analytes in the human breath, urine, sweat, and fouling gas mixtures from food. Importantly, these examples from the literature are intended to show that even with existing SERS capabilities, cheminformatics-driven applications are emerging and becoming increasingly indispensable for rapid, point-of-care applications with comprehensive data integration.

Reviewer: 3
Comments:
The Outlook by Tan and coworkers is a comprehensive overview of how the SERS technique can be more broadly employed and used by combining physical experimentation with increasingly important automation, computation, and machine learning techniques. As expected for this type of article, the authors do an excellent job of putting the topic in context and heavily employ their perspective on the current state-of-the-art and outlook. While well written, the article is going to be especially attractive to experts and users in this area, but it's less clear how comprehensible this outlook will be to a general reader. While SERS is an oft-used technique, it may be worthwhile to include a short section in the beginning of the piece describing exactly what the technique is and what it measures, and the data produced. This would go a long way to help a general reader to understand the value and importance of this work.

We thank the reviewer for the positive appraisal and constructive feedback. In this revision, we have edited the manuscript (highlighted in cyan) according to the reviewer's comment:

To improve accessibility for a broader scientific audience, we have included a brief explanatory section at the beginning of the manuscript that outlines the fundamental principles of SERS. It provides (1) a foundational overview of SERS as a vibrational spectroscopy technique and (2) explains the types of molecules SERS can detect, and (3) the spectral features it generates and how these data reveal both molecular structure and the surrounding chemical environment. This context is intended to equip general readers with the necessary background to appreciate the value of SERS and its integration with cheminformatics, automation, and machine learning. This addition will enhance the article's accessibility without detracting from its depth for more specialized readers.

The revised introduction paragraph on **page 3** reads: "Surface-enhanced Raman scattering (SERS) is a powerful spectroscopic technique that amplifies the weak Raman signals of molecules adsorbed on plasmonic nanostructures.[1-3] This signal enhancement stems from localized surface plasmon resonances, which refer to the coherent oscillations of conduction electrons excited at nanostructured interfaces, typically composed of noble metals like gold or silver.[1-3] These resonances generate intense electromagnetic fields that boost Raman scattering intensities by factors exceeding 10^6, producing sharp spectral peaks corresponding to molecular vibrational modes and serve as unique chemical fingerprints.[1-3] As a result, SERS is widely employed for ultrasensitive detection of diverse molecular species, including small organics, biological analytes, environmental pollutants, and even single biomolecules such as DNA and proteins.[1-3] In addition, the high-veracity SERS spectra not only reflect molecular structure but also respond sensitively to the local

chemical environment, including surface interactions, solvation, and binding configurations.[1-7] Overall, SERS enables ultratrace chemical detection with high sensitivity and specificity, offering qualitative and quantitative insights that make it a cornerstone of modern chemical analysis across a wide range of scientific disciplines.[8-12]"

Reviewer: 4

Please find below my comments:

We thank the reviewer for his/her thorough review and constructive feedback. In this revision, we have edited the manuscript (highlighted in grey) according to the reviewer's comments:

• The introduction requires revision, with an emphasis on lacking appropriate references. Examples includes where the authors state "Moreover, recent advances in both methodology and hardware have dramatically increased the volume and complexity of SERS data generated globally." (i.e., citations are needed for specific methodology and hardware) and where the authors also state "Currently, cheminformatics is employed for tasks such as peak identification, spectral noise reduction, multivariate statistical analysis, and pattern recognition, all of which support the identification of unknown chemicals." (i.e., similarly, citations are needed).

We thank the reviewer for highlighting this and have revised the introduction to include appropriate citations.
The revised paragraph **on page 4** reads: "In recent years, cheminformatics has played a pivotal role in addressing the challenges of managing, analyzing, and interpreting large volumes of SERS data.[23-27] By definition, cheminformatics integrates chemistry, computer science, and information technology, and uses advanced computational methods to store, index, manage, and analyze chemical datasets. These capabilities enable researchers to extract meaningful insights from raw spectral data, tasks that were previously impossible using traditional data analysis methods.[28-30] Currently, cheminformatics is employed for tasks such as peak identification, spectral noise reduction, multivariate statistical analysis, and pattern recognition, all of which support the identification of unknown chemicals and mixtures.[4, 6, 31-36] Moreover, it facilitates the integration of SERS data with complementary techniques, such as NMR, enabling more comprehensive multimodal chemical analysis.[31, 33, 37-43]"

The references added are as follows:

(23) Yi, J.; You, E.-M.; Liu, G.-K.; Tian, Z.-Q. AI–nano-driven surface-enhanced Raman spectroscopy for marketable technologies. *Nature nanotechnology* **2024**, *19* (12), 1758-1762.
(24) Simas, M. V.; Davis Jr, G. A.; Hati, S.; Pu, J.; Goodpaster, J. V.; Sardar, R. Anisotropically Shaped Plasmonic WO3–x Nanostructure-Driven Ultrasensitive SERS Detection and Machine Learning-Based Differentiation of Nitro-Explosives. *ACS Applied Materials & Interfaces* **2025**.

(25) Ding, Z.; Wang, C.; Song, X.; Li, N.; Zheng, X.; Wang, C.; Su, M.; Liu, H. Strong π-Metal Interaction Enables Liquid Interfacial Nanoarray–Molecule Co-assembly for Raman Sensing of Ultratrace Fentanyl Doped in Heroin, Ketamine, Morphine, and Real Urine. *ACS Applied Materials & Interfaces* **2023**, *15* (9), 12570-12579.

(26) Simas, M. V.; Olaniyan, P. O.; Hati, S.; Davis Jr, G. A.; Anspach, G.; Goodpaster, J. V.; Manicke, N. E.; Sardar, R. Superhydrophobic Surface Modification of Polymer Microneedles Enables Fabrication of Multimodal Surface-Enhanced Raman Spectroscopy and Mass Spectrometry Substrates for Synthetic Drug Detection in Blood Plasma. *ACS Applied Materials & Interfaces* **2023**, *15* (40), 46681-46696.

(27) Tang, J.-W.; Mou, J.-Y.; Chen, J.; Yuan, Q.; Wen, X.-R.; Liu, Q.-H.; Liu, Z.; Wang, L. Discrimination of Benign and Malignant Thyroid Nodules through Comparative Analyses of Human Saliva Samples via Metabolomics and Deep-Learning-Guided Label-free SERS. *ACS Applied Materials & Interfaces* **2025**.

(28) Pelton, J. M.; Hochuli, J. E.; Sadecki, P. W.; Katoh, T.; Suga, H.; Hicks, L. M.; Muratov, E. N.; Tropsha, A.; Bowers, A. A. Cheminformatics-Guided Cell-Free Exploration of Peptide Natural Products.
Journal of the American Chemical Society **2024**, *146* (12), 8016-8030. DOI: 10.1021/jacs.3c11306.

(29) Keith, J. A.; Vassilev-Galindo, V.; Cheng, B.; Chmiela, S.; Gastegger, M.; Müller, K.-R.; Tkatchenko, A. Combining Machine Learning and Computational Chemistry for Predictive Insights Into Chemical Systems. *Chemical Reviews* **2021**, *121* (16), 9816-9872. DOI: 10.1021/acs.chemrev.1c00107.

(30) Lee, M.-L.; Farag, S.; Del Cid, J. S.; Bashore, C.; Hallenbeck, K. K.; Gobbi, A.; Cunningham, C. N. Identification of Macrocyclic Peptide Families from Combinatorial Libraries Containing Noncanonical
Amino Acids Using Cheminformatics and Bioinformatics Inspired Clustering. *ACS Chemical Biology* **2023**, *18* (6), 1425-1434. DOI: 10.1021/acschembio.3c00159.

(31) Bajomo, M. M.; Ju, Y.; Zhou, J.; Elefterescu, S.; Farr, C.; Zhao, Y.; Neumann, O.; Nordlander, P.; Patel, A.; Halas, N. J. Computational chromatography: A machine learning strategy for demixing individual chemical components in complex mixtures. *Proceedings of the National Academy of Sciences* **2022**, *119* (52), e2211406119. DOI: doi:10.1073/pnas.2211406119.

(32) Zou, Z.; Zhang, Y.; Liang, L.; Wei, M.; Leng, J.; Jiang, J.; Luo, Y.; Hu, W. A deep learning model for predicting selected organic molecular spectra. *Nature Computational Science* **2023**, *3* (11), 957-964.

(33) Wu, S.; Zhang, Y.; He, C.; Luo, Z.; Chen, Z.; Ye, J. Self-Supervised Learning for Generic Raman Spectrum Denoising. *Analytical Chemistry* **2024**, *96* (44), 17476-17485.

(34) Leong, S. X.; Leong, Y. X.; Tan, E. X.; Sim, H. Y. F.; Koh, C. S. L.; Lee, Y. H.; Chong, C.; Ng, L. S.; Chen, J. R. T.; Pang, D. W. C. Noninvasive and point-of-care surface-enhanced Raman scattering (SERS)based breathalyzer for mass screening of coronavirus disease 2019 (COVID-19) under 5 min. *ACS nano* **2022**, *16* (2), 2629-2639.

(35) Kao, Y.-C.; Han, X.; Lee, Y. H.; Lee, H. K.; Phan-Quang, G. C.; Lay, C. L.; Sim, H. Y. F.; Phua, V. J. X.; Ng, L. S.; Ku, C. W. Multiplex surface-enhanced Raman scattering identification and quantification of urine metabolites in patient samples within 30 min. *ACS nano* **2020**, *14* (2), 2542-2552.
(36) Ehrentreich, F.; Sümmchen, L. Spike removal and denoising of Raman spectra by wavelet transform methods. *Analytical chemistry* **2001**, *73* (17), 4364-4373.
(37) Ju, Y.; Neumann, O.; Bajomo, M.; Zhao, Y.; Nordlander, P.; Halas, N. J.; Patel, A. Identifying SurfaceEnhanced Raman Spectra with a Raman Library Using Machine Learning. *ACS Nano* **2023**, *17* (21), 2125121261. DOI: 10.1021/acsnano.3c05510.
(38) Garg, A.; Nam, W.; Wang, W.; Vikesland, P.; Zhou, W. In Situ Spatiotemporal SERS Measurements and Multivariate Analysis of Virally Infected Bacterial Biofilms Using Nanolaminated Plasmonic Crystals.
ACS Sensors **2023**, *8* (3), 1132-1142. DOI: 10.1021/acssensors.2c02412.
(39) Shin, H.; Choi, B. H.; Shim, O.; Kim, J.; Park, Y.; Cho, S. K.; Kim, H. K.; Choi, Y. Single test-based diagnosis of multiple cancer types using Exosome-SERS-AI for early stage cancers. *Nature Communications* **2023**, *14* (1), 1644. DOI: 10.1038/s41467-023-37403-1.
(40) Lee, W.; Kang, B.-H.; Yang, H.; Park, M.; Kwak, J. H.; Chung, T.; Jeong, Y.; Kim, B. K.; Jeong, K.H. Spread spectrum SERS allows label-free detection of attomolar neurotransmitters. *Nature Communications* **2021**, *12* (1), 159. DOI: 10.1038/s41467-020-20413-8.
(41) Rodriguez-Nieves, A. L.; Taylor, M. L.; Wilson, R.; Eldridge, B. K.; Nawalage, S.; Annamer, A.; Miller, H. G.; Alle, M. R.; Gomrok, S.; Zhang, D. Multiplexed Surface Protein Detection and Cancer Classification Using Gap-Enhanced Magnetic–Plasmonic Core–Shell Raman Nanotags and Machine Learning Algorithm. *ACS Applied Materials & Interfaces* **2024**, *16* (2), 2041-2057.
(42) Das, S.; Saxena, K.; Tinguely, J.-C.; Pal, A.; Wickramasinghe, N. L.; Khezri, A.; Dubey, V.; Ahmad, A.; Perumal, V.; Ahmad, R. SERS nanowire chip and machine learning-enabled classification of wild-type and antibiotic-resistant bacteria at species and strain levels. *ACS Applied Materials & Interfaces* **2023**, *15* (20), 24047-24058.
(43) Jiang, H.; Zhang, Y.; Zhang, L.; Liu, L.; Wang, H.; Wang, Y.; Chen, M. Comprehensive Serum Analysis via an AI-Assisted Magnetically Driven SERS Platform for the Diagnosis and Etiological Differentiation of Childhood Epilepsy. *ACS Applied Materials & Interfaces* **2025**, *17* (8), 11731-11741.

• The authors focus heavily on molecular identification in complex mixtures throughout the manuscript. While this is certainly a relevant application, SERS can be useful for many other applications that don't involve unknown structures or complex mixtures of unknown composition. If the authors are proposing an outlook for SERS and cheminformatics generally, a broader view should be taken, including relevant literature and prospective views of the future vision in a more diverse, widespread manner.

We thank the reviewer for this question. We agree that SERS applications go beyond molecular identification in complex mixtures. In this Outlook, we highlighted diverse real-life applications in sections 2.2 and 2.3, including real-life reaction monitoring, surface chemistry investigations, mechanistic studies, environmental pollutant detection, and food fouling monitoring. In each example, we discuss cheminformatics approaches suited to these broader applications to illustrate the diversity of use cases.

• Regarding the four pillars outlined by the authors, the fourth pillar seems very broad, encompassing both automation of physical laboratory measurements via robotics with AI-driven data mining in a single pillar. I would propose these topics could be addressed separately.

We appreciate the reviewer's thoughtful suggestion. However, we have intentionally chosen to present automation and AI-driven data mining within a unified pillar because they operate in tandem toward a common objective: expanding the chemical space accessible by SERS. Automation enables the high-throughput, reproducible generation of complex spectral datasets, while AI and data mining extract historic spectral data that has already been published. These domains are inherently synergistic. Automation feeds the pipeline with high-quality data, and AI closes the loop by interpreting patterns, identifying gaps, and guiding subsequent experiments. Separating them would dilute this interplay and obscure the fact that their value is maximized when used together as part of a tightly integrated, closed-loop discovery framework. By framing them jointly, we emphasize their mutual reinforcement and highlight their collective role in advancing a scalable, data-centric vision for SERS-based chemical discovery.

We included this justification in section 2.4, on **page 30**: "In this section, we frame automation and AI-driven data mining as a unified pillar, reflecting their synergistic role in expanding the chemical space accessible by SERS. Automation generates high-throughput, reproducible spectral data, while AI mines existing datasets, identifies patterns and gaps, and guides future experiments. We emphasize that their value is maximized in a tightly integrated, closed-loop discovery framework, where each reinforces the other to enable scalable, data-centric chemical discovery."

• Looking at the list of high impact applications the authors predict advances in cheminformatics and SERS could enable (Pg 4 lines 46-54), I'm not clear on why the authors choose to focus on targeting "multiplex detection of stereoisomers and alkyl substituted analogs " via SERS, given that existing analytical methods routinely solve this challenge. Could the authors expand on why this is a high impact area where SERS is uniquely

positioned to make novel impact? The associated discussion later (Pg 15) needs to be addressed, including why this is a problem SERS is particularly well-positioned to address.

We thank the reviewer for this point. We clarified that while conventional techniques such as NMR or chiral chromatography can resolve stereoisomers and alkyl-substituted analogs under controlled conditions, these methods often require labor-intensive workflows, sample preparation and derivatization, or specific separation steps, especially when multiple structurally similar species are present in complex or dynamic environments. Multiplexed detection of such subtle molecular variants, especially in real-time or in situ settings, remains a significant analytical challenge.

Hence, SERS, especially when combined with cheminformatics, is uniquely positioned to contribute to this space. This is mainly because SERS is highly sensitive to subtle variations in molecular symmetry, conformation, and electronic environment, which are known features influenced by stereochemistry and alkyl substitution. While these differences are often masked in complex spectra, cheminformatics and machine learning can deconvolute overlapping signals to distinguish closely related isomers or analogs. Together, SERS and data-driven modeling offers a pathway toward miniaturized, on-site and real time multiplex detection, especially valuable in point-of-care diagnostics, overcoming the current bottlenecks that conventional methods struggle to deliver in multiplex, real-time settings.

The revised sentences **on page 4** reads "We envision that future advances in the interface of SERS and cheminformatics will drive miniaturized, on-site and point-of-care applications, including (1) identifying unknown compounds in complex matrices and predicting novel reactive species in reaction mechanisms, (2) facilitating rapid multiplex detection of stereoisomers and alkylsubstituted analogs, and (3) supporting targeted -omics analyses, including metabolomics, proteomics, and genomics, complementing existing gold standard liquid chromatography–mass spectrometry and sequencing tools.[4, 41, 44-53]"

- Similarly, regarding application #3 (Pg. 4 lines 46-54), with regards to the discussion of genomics and proteomics as high impact SERS applications, could the authors please expand on this more specifically? And moreover, elaborate and clarify the cases in which SERS could become a preferred analytical method? Mapping genomes or proteomes sounds like a particularly difficult challenge with SERS and it's not clear to me in what cases SERS would be a preferred method of measurement over existing techniques in these fields. There are other perhaps more obvious advancements I could envision, such as further enhancing limits of detection using the framework the authors outline, so it would be useful to provide more detail on why specifically these envisioned applications were highlighted.

We thank the reviewer for this question. We agree that the use of SERS in genomics and proteomics is still emerging. We clarified that our emphasis lies in the potential of SERS for targeted genomic or proteomic sensing, such as single-base mutation detection, aptamer-based sensing, and structural fingerprinting of peptides or folded proteins, and cited the relevant examples. Furthermore, rather than for full-scale mapping, we highlight that SERS can support or complement existing tools.

The revised sentence **on page 4** reads "... (3) supporting targeted -omics analyses, including metabolomics, proteomics, and genomics, complementing existing gold standard liquid chromatography–mass spectrometry and sequencing tools.[4, 41, 44-53]"

- The use of molecular dynamics simulations to confirm spectral assignments certainly makes sense in some cases. In complex mixtures of unknown composition, where de novo molecular structure identification is the "holy grail," could the authors please discuss how molecular dynamics do or do not participate in their framework? I'm not clear on how useful simulations will be when the overall composition of the mixture is unknown and identifying individual components is a challenge. Also, building on this, given the work that exists in the literature for simulated Raman spectra and associated model training of known composition, the authors should identify and present the key challenges and/or limitations associated with this current state to then build a future-looking vision that is both specific and detailed.

We appreciate this request for clarification. We agree that MD simulations are not intended for full de novo structural elucidation in unknown mixtures but rather to establish structure-spectra relationships, validating candidate molecular geometries, and modeling solvation or adsorption effects that influence spectral features.
We highlighted this **on page 21**, "While molecular dynamics are not intended for full de novo structural elucidation in unknown mixtures, it is valuable in defining structure-spectra relationships, validating candidate molecular geometries, and modeling solvation or adsorption effects that influence spectral features."

Regarding the use of simulated Raman spectra to predict mixtures, we clarify that at the time of writing and submission, the recently accepted work on using simulated Raman spectra for mixture analysis (Ju et al., 2025) was not yet available in the literature. We have since included a review of the work and discussed key challenges and/or limitations associated with this current state. The revised paragraph **on page 21** reads: "In a recent work, a physics-informed analytical approach that integrates DFT-simulated Raman spectra with SERS enabled accurate identification of polycyclic aromatic hydrocarbons from contaminated soil, including those lacking experimental reference spectra.

Combining a characteristic peak extraction (CaPE) and similarity (CaPSim) algorithms, the method overcomes challenges including spectral interference and variability, demonstrating strong agreement between simulated and experimental SERS spectra for multiple polycyclic aromatic hydrocarbons. Although the method performs well on polycyclic aromatic analytes with high Raman scattering efficiencies, it is crucial to demonstrate its generalizability to analytes with low scattering efficiencies that are essential for real-world sensing applications. To achieve this, it is important to consider two factors. First, there is a significant domain gap in which simulated spectra are idealized and lack experimental artifacts such as noise, baseline drifts, and fluorescence, which can impair model transferability to weakly scattering analytes. Second, the accuracy of simulations is constrained by methodological choices (e.g., functional, basis set) and often degrades for large, flexible, or highly interactive molecules. In the context of SERS, most simulations insufficiently capture key effects such as plasmonic enhancement and molecule– surface interactions, which substantially influence spectral signatures. Moreover, the computational cost of high-fidelity simulations limits chemical diversity, resulting in datasets biased toward rigid molecules that do not reflect the complexity of real sensing targets. DFT simulations also neglect dynamic and environmental factors such as temperature and pH, which affect spectra in practice. Finally, the assumption of well-defined compositions in simulated data does not hold for complex mixtures, where spectral overlap and molecule-molecule interaction effects complicate both labeling and interpretation. These limitations highlight the need for strategies that combine simulated and experimental data using domain adaptation approaches toward generalizable in silico-based SERS sensing."

- The authors mention standard databases for different substrates and instruments. Given the body of work that exists in the literature to date for evaluating different substrates across diverse instrumentation, the authors should clarify what they are proposing differently? How is the field advancing relative to this body of work? Detailing a summary of current literature reports that then proposed a future proposed guideline is needed, including covering the topic of when a SERSbased methodology is applied across disparate analytical instrumentation, how can we robustly determine the similarity of the generated results and account for differences in instrumental design, calibration, and other key analytical features inherent to the instrument itself?

We thank the reviewer for this important point. In Section 2.1, we highlight a cheminformaticsdriven strategy to harmonize SERS results across different substrates and instruments by establishing a centralized, metadata-rich SERS database. We summarize existing inter-laboratory benchmarking efforts and propose a six-step standardized

reporting protocol that includes critical metadata, such as substrate morphology, laser wavelength, and acquisition parameters, to support this goal. These efforts lay the groundwork for an interoperable, cross-platform SERS repository capable of addressing variability in instrument design, calibration, and measurement conditions.

While commercial Raman databases exist, current SERS databases are limited in scope, covering few analytes and measured on disparate plasmonic substrates. It is therefore essential to unify existing datasets and concurrently build a comprehensive, standardized SERS database encompassing diverse analytes, lasers, substrates, and experimental conditions.
The revised sentence **on page 13 reads** "Ultimately, we envision a cheminformatics approach to harmonize SERS results across substrates and instruments through a metadata-rich, centralized database. Specifically, we outline a six-step standardized reporting protocol that captures essential metadata, such as substrate morphology, laser wavelength, and acquisition parameters, to support an interoperable SERS database that accounts for variations in instrument design and calibration."

Some of the aforementioned existing SERS databases, which we have highlighted in the outlook section 3.4 **on page 41** include:
(125) Yuan, Q.; Tang, J.-W.; Chen, J.; Liao, Y.-W.; Zhang, W.-W.; Wen, X.-R.; Liu, X.; Chen, H.-J.; Wang, L. SERS-ATB: A comprehensive database server for antibiotic SERS spectral visualization and deep-learning identification. *Environmental Pollution* **2025**, *373*, 126083.
(126) Wang, A.; Han, J.; Guo, L.; Yu, J.; Zeng, P. Database of standard Raman spectra of minerals and related inorganic crystals. *Applied Spectroscopy* **1994**, *48* (8), 959-968.

(127) Shi, H.; Wang, H.; Meng, X.; Chen, R.; Zhang, Y.; Su, Y.; He, Y. Setting up a surfaceenhanced Raman scattering database for artificial-intelligence-based label-free discrimination of tumor suppressor genes. *Analytical chemistry* **2018**, *90* (24), 14216-14221.
(128) Sherman, L. M.; Petrov, A. P.; Karger, L. F.; Tetrick, M. G.; Dovichi, N. J.; Camden, J. P. A surface-enhanced Raman spectroscopy database of 63 metabolites. *Talanta* **2020**, *210*, 120645.

- The authors proposal for developing LLMs for SERS should present a more thorough foundation that includes, for instance, what are the costs associated with developing a LLM for SERS, what specific type and level of fine tuning is required, how robust is this proposed fine tuning, and other critical information that can turn this proposed vision from a more lofty vision to a realistic aspirational goal the field should move towards.

We thank the reviewer for this question. We have expanded the section on large language models (LLMs) to discuss the practical cost and considerations involved in their development for SERS, including the need for domain-specific pretraining, fine-tuning on spectral-text corpora, data volume requirements, and computational costs. To make our vision more concrete and actionable, we also outline specific use cases such as automated spectral annotation, hypothesis generation, and spectral extraction using LLM-based tools.

The revised section **on page 35** includes "Nevertheless, developing LLMs for SERS requires domain-specific pretraining, as general-purpose models lack the specialized knowledge needed to interpret spectroscopic data accurately. Fine-tuning on spectral-text corpora is essential, yet this demands access to high-quality, well-annotated datasets that include experimental metadata, spectral features, and analyte information, resources that are often limited or inconsistently reported.[113, 114] Furthermore, the data volume requirements for such tasks are substantial, particularly given the need to handle multimodal inputs like spectra, chemical structures, and textual descriptions.[17, 111, 115] Additionally, the computational costs of training and deploying these models are significant.[111, 115] To evaluate the actionability of this strategy, we draw inspiration from previous use cases in adjacent fields such as automated spectral annotation, hypothesis generation, and spectral extraction from literature using LLM-based tools.[75, 107, 116-119]"

- Furthermore, the authors mention quantum machine learning (QML) and to make this section more impactful, the authors should provide a more thorough overview of the topic. The current description seems vague and only hints at the advantages of such techniques but fails to address the current state of the field, any challenges and/or limitations with development and subsequent implementation, and what the proposal for benchmarking of methodologies either currently is or may look like in the future.

We thank the reviewer for this question. We have revised and supplemented the additional discussion in the QML section. The updated text now includes an overview of current hybrid quantum-classical approaches for spectral prediction, examples of quantum kernel methods applied to molecular data, and a discussion of the limitations posed by noise, scalability, and hardware availability. We also propose benchmarking strategies and identify areas where QML may offer an advantage, such as in high-dimensional feature reduction or small-data scenarios common in SERS.

The revised section **on page 38** includes "Current hybrid quantum-classical approaches for spectral prediction combine quantum computing's potential for modeling molecular systems with classical ML to enhance accuracy and efficiency. Examples include quantum kernel methods

applied to molecular property prediction, where quantum circuits encode structural information for improved classification or regression performance.[72, 96, 120-124] However, these approaches face limitations due to quantum hardware noise, limited qubit counts, and scalability challenges that restrict their practical use.[96, 120, 121] Looking forward, we propose benchmarking strategies that compare quantum and classical models across relevant SERS tasks, using standardized datasets and performance metrics. Notably, QML may offer distinct advantages in high-dimensional feature reduction and small-data regimes common in SERS due to experimental constraints.[96, 120]"

- A broader question I leave wondering is how close the field is to achieving the goals outlined by the authors in this outlook. Providing additional detail on efforts currently underway and/or pointing readers to existing databases being constructed, etc. is needed.

We thank the reviewer for this question. We have added a final section summarizing ongoing efforts that align with our proposed framework, including publicly available spectral databases, open-source cheminformatics platforms. We also outline short- and mid-term milestones that would enable the field to realize the vision in this Outlook, including infrastructure needs, collaborative consortia, and data-sharing standards. The revised section **on page 41** includes "Ongoing efforts aligned with our framework, including public and commercial SERS and Raman spectral databases[22, 69, 125-130], as well as open-source cheminformatics tools such as RDKit and PySpark,[131-133] and recent advances in SERS–ML integration demonstrating proof-of-concept workflows for automated analyte identification and spectral interpretation,[4-6, 22, 34] reinforces the feasibility of our vision. Short-term milestones such as curating metadata-rich datasets, standardizing spectral formats, while mid-term goals include forming collaborative consortia, establishing data-sharing protocols, sharing molecular modeling insights, and developing cloud-based infrastructure for the centralized database. Collectively, these steps lay the foundation for cheminformatics-driven SERS research that extends beyond ML to fully leverage tools like molecular modeling and database management, ultimately pushing the limits of SERS."

oc-2025-00785q.R2

Name: Peer Review Information for "SERS Cheminformatics: Opportunities for Data-Driven Discovery and Applications"

Second Round of Reviewer Comments

Reviewer: 2

Comments to the Author

I asked several fundamental questions whethere these results can be treated as one of informatics or not.

I don't think any of answers can satisfy the questions.

As I mentioned, I think this concept is attractive, but the results are very far from satisfactory.

Author's Response to Peer Review Comments:

Dear Editor, 8th July 2025

Subject: Submission of revised manuscript

We thank you and the reviewers for the positive feedback and constructive suggestions thus far, as well as for provisionally accepting our manuscript, entitled "SERS Cheminformatics: Opportunities for Data-Driven Discovery and Applications." We are delighted to hear this positive news.

We have carefully reviewed the requested formatting changes and ensured that all submitted publication files are clean and free of any markups, highlights, colored text, or tracked change. As requested, this revision focuses solely on the specified formatting issues. We have not made any changes to the scientific content of the manuscript.

We look forward to the publication of our manuscript. We appreciate your time and consideration in reviewing our manuscript. Thank you!

On behalf of all authors,

Yours sincerely,

Xing Yi Ling

Professor

21 Nanyang Link, Chemistry and Biological Chemistry Division

School of Chemistry, Chemical Engineering and Biotechnology

Nanyang Technological University, Singapore
637371 Tel: +65-6513 2740; Email:
xyling@ntu.edu.sg
http://www3.ntu.edu.sg/home/xyling/index.html
